# Bi_2_Te_3_/Bi_2_Se_3_/Bi_2_S_3_ Cascade Heterostructure for Fast‐Response and High‐Photoresponsivity Photodetector and High‐Efficiency Water Splitting with a Small Bias Voltage

**DOI:** 10.1002/advs.202205460

**Published:** 2022-12-27

**Authors:** Chunhui Lu, Mingwei Luo, Wen Dong, Yanqing Ge, Taotao Han, Yuqi Liu, Xinyi Xue, Nan Ma, Yuanyuan Huang, Yixuan Zhou, Xinlong Xu

**Affiliations:** ^1^ Shaanxi Joint Lab of Graphene State Key Laboratory of Photon‐Technology in Western China Energy International Collaborative Center on Photoelectric Technology and Nano Functional Materials Institute of Photonics & Photon‐Technology School of Physics Northwest University Xi'an 710069 China

**Keywords:** Bi_2_Te_3_/Bi_2_Se_3_/Bi_2_S_3_ cascade heterostructure, fast response, high‐photoresponsivity, photoelectrochemical photodetector, water splitting

## Abstract

Large‐scale multi‐heterostructure and optimal band alignment are significantly challenging but vital for photoelectrochemical (PEC)‐type photodetector and water splitting. Herein, the centimeter‐scale bismuth chalcogenides‐based cascade heterostructure is successfully synthesized by a sequential vapor phase deposition method. The multi‐staggered band alignment of Bi_2_Te_3_/Bi_2_Se_3_/Bi_2_S_3_ is optimized and verified by X‐ray photoelectron spectroscopy. The PEC photodetectors based on these cascade heterostructures demonstrate the highest photoresponsivity (103 mA W^−1^ at −0.1 V and 3.5 mAW^−1^ at 0 V under 475 nm light excitation) among the previous reports based on two‐dimensional materials and related heterostructures. Furthermore, the photodetectors display a fast response (≈8 ms), a high detectivity (8.96 × 10^9^ Jones), a high external quantum efficiency (26.17%), and a high incident photon‐to‐current efficiency (27.04%) at 475 nm. Due to the rapid charge transport and efficient light absorption, the Bi_2_Te_3_/Bi_2_Se_3_/Bi_2_S_3_ cascade heterostructure demonstrates a highly efficient hydrogen production rate (≈0.416 mmol cm^−2^ h^−1^ and ≈14.320 µmol cm^−2^ h^−1^ with or without sacrificial agent, respectively), which is far superior to those of pure bismuth chalcogenides and its type‐II heterostructures. The large‐scale cascade heterostructure offers an innovative method to improve the performance of optoelectronic devices in the future.

## Graphical abstract

The centimeter‐scale bismuth chalcogenides based cascade heterostructure are synthesized by vapor phase deposition methods. The Bi_2_Te_3_/Bi_2_Se_3_/Bi_2_S_3_ heterostructure photodetectors demonstrate a high photoresponsivity (103 mA W^−1^ at −0.1 V and 3.5 mAW^−1^ at 0 V) and a fast response (≈8 ms) at 475 nm. The cascade heterostructure also shows a high hydrogen production rate (0.416 mmol cm^−2^ h^−1^).

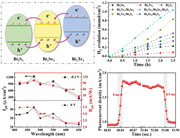

## 1 Introduction

The conversion of solar energy into electric and chemical energy by photoelectrochemical (PEC) devices has received widespread attention due to the promising prospects in solving energy‐shortage and environment‐pollution.^[^ ^1^ ^]^ The key challenge in PEC devices is to develop photoelectrode materials with fast response and high photocurrent density under a small bias or without a bias voltage.^[^ ^2^ ^]^ In the past few decades, n‐type semiconductors such as TiO_2_ and ZnO^[^ ^3^ ^]^ have been widely investigated as photoanodes in PEC devices due to their strong redox capability. However, the low conductivity, limited carrier lifetime, rapid carrier recombination rate, and large bandgap in UV band only have prevented the energy conversion efficiency by these wide bandgap semiconductors.^[^ ^4^ ^]^ In this regard, hunting for advanced materials such as visible‐light‐active two‐dimensional (2D) materials^[^ ^5^ ^]^ is desirable for PEC devices due to their narrow bandgap, strong light‐matter interaction, and high carrier mobility.

As typical 2D materials, bismuth chalcogenides with a general structure Bi_2_X_3_ (X = Te, Se, and S) are attractive and promising candidates for visible‐light‐driven PEC devices due to the narrow bandgap and environmental friendliness. Among them, Bi_2_Se_3_ and Bi_2_Te_3_ are the most studied topological insulators with the metallic Dirac surface states,^[^ ^6^ ^]^ which are advantageous to the electron interface transmission. Furthermore, both Bi_2_Se_3_ and Bi_2_Te_3_ show a superior electrical conductivity and a small bandgap as well as a high surface mobility (10^4^ cm^2^ V^−1^ s^−1^).^[^ ^7^ ^]^ In contrast, Bi_2_S_3_ belongs to a typical n‐type semiconductor with a relatively high photon‐electron conversion efficiency (≈5%), a high absorption coefficient (10^4^–10^5^ cm^−1^), and a narrow bandgap (≈1 eV).^[^ ^8^ ^]^ Despite the strong and wide‐band light absorption by bismuth chalcogenides, the rapid electron‐hole pair recombination in them still slows down the interfacial kinetics and reduces the conversion efficiency in the photodetection and water splitting in PEC performance.

To solve the above problems, Bi_2_X_3_‐based heterostructures such as Bi_2_X_3_/SnS_2_,^[^ ^5^, ^9^ ^]^ Bi_2_S_3_/BiVO_4_,^[^ ^10^ ^]^ Bi_2_Se_3_/TiO_2_,^[^ ^11^ ^]^ and Bi_2_Se_3_/MoS_2_^[^ ^12^ ^]^ have been constructed as a strategy to improve the photocatalytic activity due to the rapid carrier migration at the interface. This type‐II heterostructure with a staggered band structure can effectively reduce the severe charge recombination by the built‐in electric field,^[^ ^13^ ^]^ which is of great significance for PEC water splitting and PEC‐type photodetectors. However, the poor interfacial contact and charge traps at the interface seriously hinder the charge flow trajectory and decrease the charge transfer efficiency. Considering the similar crystal structure and conducting surface state, the Bi_2_X_3_ heterostructures are easy to form intimate interface and beneficial for charge transfer. As such, the heterostructure formation among 2D Bi_2_X_3_ provides an efficient strategy for realizing highly efficient PEC performance. Moreover, recent works on cascade heterostructures such as ZnO/CdS/CdSe, ZnO/CdS/PbS, and MoS_2_/WS_2_/WSe_2_/Si^[^ ^14^ ^]^ suggest that the multi‐staggered band alignment can further accelerate the electron‐hole flow fluently and improve the carrier separation efficiently. Recently, type‐II Bi_2_S_3_/Bi_2_Se_3_ heterostructure was fabricated as anode materials for hybrid capacitors due to the efficient charge transport.^[^ ^15^ ^]^ Bi_2_Te_3_/Bi_2_Se_3_ heterostructure was also constructed to improve the thermoelectric performance.^[^ ^16^ ^]^ However, despite the efficient control in size and atomic uniformity of 2D Bi_2_X_3_ films by a vapor deposition method,^[^ ^5^, ^17^ ^]^ the synthesis of the Bi_2_Te_3_/Bi_2_Se_3_/Bi_2_S_3_ cascade heterostructure and the optimization of band alignment are still in challenge.

Herein, a sequential vapor phase deposition method is optimized to synthesize Bi_2_Te_3_/Bi_2_Se_3_/Bi_2_S_3_ thin films with cascade band alignment. The multiple staggered bandgap has been verified by X‐ray photoelectron spectroscopy (XPS) and UV–vis absorption spectra. The PEC‐type photodetectors based on these cascade heterostructures demonstrate a broadband photoresponse with the maximum photoresponsivity of 103 and 3.5 mAW^−1^ at −0.1 and 0 V, respectively. Due to the high incident photon‐to‐current efficiency (IPCE ≈27.04%), the Bi_2_Te_3_/Bi_2_Se_3_/Bi_2_S_3_ cascade heterostructure also reaches unprecedented hydrogen (H_2_) production rates of 0.416 mmol cm^−2^ h^−1^ and 14.320 µmol cm^−2^ h^−1^ in the sacrificial agent and pure water, respectively. This study reveals the great potentials of cascade heterostructures based on 2D materials for high‐performance PEC devices.

## 2 Results and Discussion

**Figure 1:**
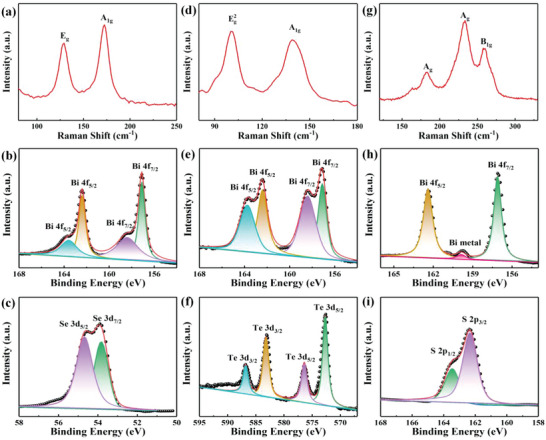
a) Raman spectrum, and XPS spectra of b) Bi 4f core level and c) Se 3d core level of Bi_2_Se_3_ film; d) Raman spectrum, and XPS spectra of e) Bi 4f core level and f) Te 3d core level of Bi_2_Te_3_ film; g) Raman spectrum, and XPS spectra of h) Bi 4f core level and i) S 2p core level of Bi_2_S_3_ film.

Chemical/physical vapor deposition (CVD/PVD) is a facile strategy to fabricate large‐scale nanofilms. The uniform Bi_2_Se_3_ film in a centimeter scale (Figure S1a, Supporting Information) was synthesized by a PVD method (see Experimental Section for details). The characteristic Raman peaks in **Figure** **1**a were found near 128.3 and 172.0 cm^−1^, which match well the *E*_g_ and *A*_1g_ vibration modes of Bi_2_Se_3_.^[^ ^12^ ^]^ To further analyze the surface compositions and element states of the as‐grown materials, the XPS spectra of Bi 4f and Se 3d signals in the Bi_2_Se_3_ film were measured as shown in Figure 1b,c. There are two main peaks at 157.1 eV of Bi 4f_7/2_ and 162.4 eV of Bi 4f_5/2_ from Bi_2_Se_3_. As compared, two tiny peaks at 158.4 and 163.6 eV in Figure 1b are from Bi_2_O_3_, which may be due to the natural oxidation.^[^ ^18^ ^]^ From the XPS spectrum of Se 3d core level, the Se 3d_5/2_ (≈54.7 eV) and Se 3d_7/2_ (≈53.8 eV) belong to the Se^2−^ valence state. This analysis further confirms the successful formation of Bi_2_Se_3_ films. Due to the similar physical and chemical properties of Bi_2_Se_3_ and Bi_2_Te_3_, the same parameters were also used to grow Bi_2_Te_3_ film by a PVD method with the morphology shown in Figure S1b (Supporting Information). The characteristic Raman peaks near 99.9 and 139.1 cm^−1^ in Figure 1d are the $E_{g}^{2}$ and A_1g_ vibration modes of Bi_2_Te_3_.^[^ ^19^ ^]^ The Bi 4f core‐level spectrum also exhibits four main peaks (162.4, 163.7, 157.1, and 158.4 eV) from Bi^3+^ as shown in Figure 1e. The four peaks of Te 3d are 3d_5/2_ (≈572.7 and 576.4 eV) and 3d_3/2_ (≈583.2 and 586.8 eV) in Figure 1f. The synthesized Bi_2_Te_3_ film with Bi and Te oxidation states may be due to the long‐term air exposure in the measurement.^[^ ^6^ ^]^ Compared with the PVD method, the Bi_2_S_3_ film was synthesized by a CVD method to avoid the high decomposition under high temperature (see Experimental Section for details). The photograph and scanning electron microscopy (SEM) characterization of Bi_2_S_3_ film suggests that the nanosheets are deposited and stacked a continuous and uniform film as shown in Figure S1c (Supporting Information). The Raman spectrum of Bi_2_S_3_ in Figure 1g shows the A_g_ vibration modes near 183.2 and 233.6 cm^−1^, and the B_1g_ vibration mode near 259.2 cm^−1^.^[^ ^20^ ^]^ The XPS characterization in Figure 1h demonstrates that the peaks (162.4 and 157.1 eV) of Bi 4f are from Bi^3+^. The small peak at 159.8 eV is from Bi metal, which is caused by a bit precipitation of metal bismuth in the CVD reaction process. From the S 2p core‐level spectrum in Figure 1i, the peaks of S 2p_1/2_ and 2p_3/2_ energy levels are observed near 163.46 and 162.35 eV, respectively. Meanwhile, hexagonal phase Bi_2_Se_3_, hexagonal phase Bi_2_Te_3_, and orthorhombic phase Bi_2_S_3_ have been confirmed by X‐ray diffraction spectroscopy as shown in Figure S2 (Supporting Information).

**Figure 2:**
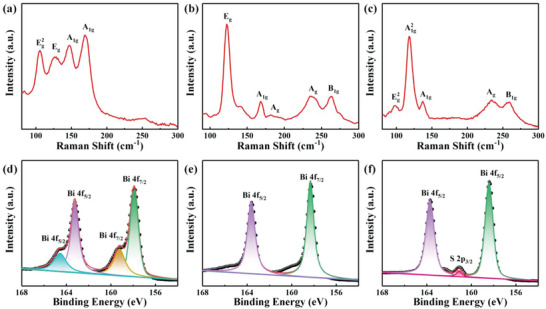
a–c) Raman spectra and d–f) XPS spectra of Bi 4f core level in the Bi_2_Te_3_/Bi_2_Se_3_, Bi_2_Se_3_/Bi_2_S_3_, and Bi_2_Te_3_/Bi_2_S_3_, respectively.

To optimize the heterostructure formation and confirm the band alignment, Bi_2_X_3_ heterostructures (Bi_2_Te_3_/Bi_2_Se_3_, Bi_2_Se_3_/Bi_2_S_3_, and Bi_2_Te_3_/Bi_2_S_3_) were first prepared by a two‐step vapor phase deposition method. The uniform Bi_2_X_3_ heterostructure films are observed from photographs and SEM images as shown in Figure S1d–f (Supporting Information) and the top layer films were successfully deposited onto the bottom layer films. From the Raman spectrum of Bi_2_Te_3_/Bi_2_Se_3_ in **Figure** **2**a, there are four vibration modes such as E_g_ (126.9 cm^−1^) and A_1g_ (169.3 cm^−1^) from Bi_2_Se_3_ and $E_{g}^{2}$ (105.7 cm^−1^) and A_1g_ (146.7 cm^−1^) from Bi_2_Te_3_. Similarly, the A_g_ (≈186.2 and 235.2 cm^−1^) and B_1g_ (≈263.7 cm^−1^) vibration modes from Bi_2_S_3_ are also displayed in Figure 2b,c, which also confirms the formation of Bi_2_Se_3_/Bi_2_S_3_ and Bi_2_Te_3_/Bi_2_S_3_, respectively. It is worth noting that the characteristic peaks have an obvious red‐shift or blue‐shift in these heterostructures. This is due to the interlayer coupling when the heterostructure interface is formed, which is the direct evidence of van der Waals heterostructures.^[^ ^21^ ^]^

To determine the band offset parameters, high‐resolution XPS is employed to evaluate the valence band offset (Δ*E*_v_) at the Bi_2_X_3_ interfaces. This offset is the energy difference of the Bi core levels between the heterostructure and the single component. In the Bi_2_Te_3_/Bi_2_Se_3_ heterostructure, four main peaks of Bi 4f_5/2_ and Bi 4f_7/2_ are 164.6, 163.2, 159.2, and 157.9 eV, respectively, as shown in Figure 2d. Similarly, the Bi core levels of Bi_2_Se_3_/Bi_2_S_3_ (163.6 eV of Bi 4f_5/2_ and 158.3 eV of 4f_7/2_) and Bi_2_Te_3_/Bi_2_S_3_ (163.7 eV of Bi 4f_5/2_, 158.4 eV of Bi 4f_7/2_, and 161.1 eV of S 2p_3/2_) heterostructures are also investigated as shown in Figure 2e,f. The corresponding Te, Se, and S core levels are fitted and shown in Figure S3 (Supporting Information). These core levels in the heterostructures have a shift toward higher or lower binding energy compared with those of pure Bi_2_Te_3_, Bi_2_Se_3_, and Bi_2_S_3_, indicating the interfacial carrier redistribution when the heterostructure is formed.

**Figure 3:**
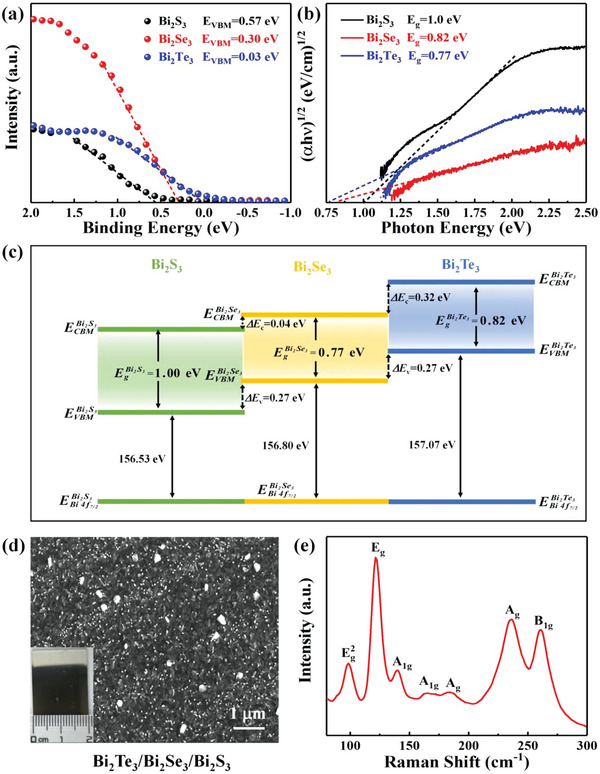
a) Valence band spectra and b) Tauc plots of Bi_2_X_3_ films; c) Schematic diagram of band arrangement of the Bi_2_Te_3_/Bi_2_Se_3_/Bi_2_S_3_ cascade heterostructure; d) SEM image with the photograph shown in the inset, and e) Raman spectrum of the Bi_2_Te_3_/Bi_2_Se_3_/Bi_2_S_3_ cascade heterostructure.

The valence band spectra of Bi_2_S_3_, Bi_2_Se_3,_ and Bi_2_Te_3_ were also measured by XPS to calculate the band arrangement structure at the interface. As shown in **Figure** **3**a, the maximum valence bands (VBM) of the Bi_2_S_3_, Bi_2_Se_3_, and Bi_2_Te_3_ films are 0.57, 0.30, and 0.03 eV, respectively. The valence band offset parameters (Δ*E*_V_) of the Bi_2_Se_3_/Bi_2_S_3_, Bi_2_Te_3_/Bi_2_Se_3_, and Bi_2_Te_3_/Bi_2_S_3_ films can be calculated as follows:^[^ ^5^, ^22^ ^]^

(1) $$\begin{array}{ccc} \Delta E_{V1} & = & \left(E_{\mathrm{Bi}4f_{7/2}}^{{\mathrm{Bi}}_{2}S_{3}}-E_{\mathrm{VBM}}^{{\mathrm{Bi}}_{2}S_{3}}\right)+\left(E_{\mathrm{Bi}4f_{7/2}}^{{\mathrm{Bi}}_{2}{\mathrm{Se}}_{3}/{\mathrm{Bi}}_{2}S_{3}}-E_{\mathrm{Bi}4f_{7/2}}^{{\mathrm{Bi}}_{2}{\mathrm{Se}}_{3}/{\mathrm{Bi}}_{2}S_{3}}\right)\\ & & -\left(E_{\mathrm{Bi}4f_{7/2}}^{{\mathrm{Bi}}_{2}{\mathrm{Se}}_{3}}-E_{\mathrm{VBM}}^{{\mathrm{Bi}}_{2}{\mathrm{Se}}_{3}}\right) \end{array}$$

(2) $$\begin{array}{ccc} \Delta E_{V2} & = & \left(E_{\mathrm{Bi}4f_{7/2}}^{{\mathrm{Bi}}_{2}{\mathrm{Se}}_{3}}-E_{\mathrm{VBM}}^{{\mathrm{Bi}}_{2}{\mathrm{Se}}_{3}}\right)+\left(E_{\mathrm{Bi}4f_{7/2}}^{{\mathrm{Bi}}_{2}{\mathrm{Te}}_{3}/{\mathrm{Bi}}_{2}{\mathrm{Se}}_{3}}-E_{\mathrm{Bi}4f_{7/2}}^{{\mathrm{Bi}}_{2}{\mathrm{Te}}_{3}/{\mathrm{Bi}}_{2}{\mathrm{Se}}_{3}}\right)\\ & & -\left(E_{\mathrm{Bi}4f_{7/2}}^{{\mathrm{Bi}}_{2}{\mathrm{Te}}_{3}}-E_{\mathrm{VBM}}^{{\mathrm{Bi}}_{2}{\mathrm{Te}}_{3}}\right) \end{array}$$

(3) $$\begin{array}{ccc} \Delta E_{V3} & = & \left(E_{\mathrm{Bi}4f_{7/2}}^{{\mathrm{Bi}}_{2}S_{3}}-E_{\mathrm{VBM}}^{{\mathrm{Bi}}_{2}S_{3}}\right)+\left(E_{\mathrm{Bi}4f_{7/2}}^{{\mathrm{Bi}}_{2}{\mathrm{Te}}_{3}/{\mathrm{Bi}}_{2}S_{3}}-E_{\mathrm{Bi}4f_{7/2}}^{{\mathrm{Bi}}_{2}{\mathrm{Te}}_{3}/{\mathrm{Bi}}_{2}S_{3}}\right)\\ & & -\left(E_{\mathrm{Bi}4f_{7/2}}^{{\mathrm{Bi}}_{2}{\mathrm{Te}}_{3}}-E_{\mathrm{VBM}}^{{\mathrm{Bi}}_{2}{\mathrm{Te}}_{3}}\right) \end{array}$$

According to the experimental data, the Δ*E*_V1_ value of Bi_2_Se_3_/Bi_2_S_3_ heterostructure is −0.27 eV, and the Δ*E*_V2_ value of Bi_2_Te_3_/Bi_2_Se_3_ heterostructure is −0.27 eV, and the Δ*E*_V3_ value of Bi_2_Te_3_/Bi_2_S_3_ heterostructure is −0.54 eV. In order to measure the bandgap (*E*_g_) of Bi_2_X_3_, the Tauc plots of Bi_2_S_3_, Bi_2_Se_3_, and Bi_2_Te_3_ in Figure 3b are derived from the absorption spectroscopy (Figure S4, Supporting Information). The following relationship exists between the bandgap and the photon energy:

(4) $$\alpha h\nu =A{\left(h\nu -E_{g}\right)}^{1/2}$$

**Table 1:** Performance comparison of PEC photodetectors based on Bi_2_Te_3_/Bi_2_Se_3_/Bi_2_S_3_ and other previously reported advanced materials under a bias voltage

Material	Type	Light	Bias	*R*_ph_ [mA W^−1^]	*t*_res_/*t*_rec_ [ms]	Ref
Bi_2_Te_3_/Bi_2_Se_3_/Bi_2_S_3_	PEC (0.1 m Na_2_S/0.02 m Na_2_SO_3_)	475 nm	−0.1 V	103	8/6.9	This work
PbO	PEC (0.01 KOH)	475 nm	0.4 V	≈0.725	100/100	[38]
GDY	PEC (0.1 m KOH)	365 nm	0.6 V	0.05067	–	[39]
Bi_2_O_2_S	PEC (1.0 m KOH)	365 nm	0.6 V	13.0	10/45	[40]
InSe	PEC (0.2 m KOH)	Sun	1 V	0.0033	5000/‐	[41]
GeSe	PEC (0.1 m KOH)	Sun	0.3 V	0.0436–0.076	200/300	[1b]
SnS	PEC (0.1 m Na_2_SO_4_)	Sun	0.6 V	0.06	300/‐	[36a]
SnS	PEC (0.5 m Na_2_SO_4_)	Sun	0.4 V	0.0182	600/300	[36b]
PbSe	PEC (0.1 m KOH)	Sun	0.2 V	0.01237	120/130	[42]
SnS_2_/Bi_2_Se_3_	PEC (−0.1 m Na_2_SO_3_)	Sun	−0.1 V	2.43	–	[43]
SnS/SnSe_2_	PEC (0.5 m Na_2_SO_4_)	Sun	0.6 V	0.28	9.1/97.9	[37a]
Te@Se	PEC (0.5 m Na_2_SO_4_)	Sun	0.6 V	0.099	520/‐	[37b]

Here, *α, h, ν*, and *A* are the absorption coefficient, Planck's constant, optical frequency, and proportionality constant, respectively. Based on the calculation of Equation 4, the bandgaps of the Bi_2_S_3_, Bi_2_Se_3,_ and Bi_2_Te_3_ films are 1.00, 0.82, and 0.77 eV, respectively. Therefore, the corresponding conduction band offset parameters (Δ*E*_c_) of Bi_2_X_3_ heterostructures can be calculated by the following Equations:

(5) $$\Delta E_{C1}=E_{g}^{{\mathrm{Bi}}_{2}S_{3}}+\Delta E_{V1}-E_{g}^{{\mathrm{Bi}}_{2}{\mathrm{Se}}_{3}}$$

(6) $$\Delta E_{C2}=E_{g}^{{\mathrm{Bi}}_{2}{\mathrm{Se}}_{3}}+\Delta E_{V2}-E_{g}^{{\mathrm{Bi}}_{2}{\mathrm{Te}}_{3}}$$

(7) $$\Delta E_{C3}=E_{g}^{{\mathrm{Bi}}_{2}S_{3}}+\Delta E_{V3}-E_{g}^{{\mathrm{Bi}}_{2}{\mathrm{Te}}_{3}}$$

According to the experimental data, the Δ*E*_C1_ value of Bi_2_Se_3_/Bi_2_S_3_ is −0.04 eV, and the Δ*E*_C2_ value of Bi_2_Te_3_/Bi_2_Se_3_ is ‐0.32 eV, and the Δ*E*_C3_ value of Bi_2_Te_3_/Bi_2_S_3_ is −0.36 eV. Based on these results, the schematic diagram of the band arrangement of the Bi_2_Te_3_/Bi_2_Se_3_/Bi_2_S_3_ cascade heterostructure is obtained as shown in Figure 3c. This band alignment of cascade heterostructure suggests the synchronization of the electron‐hole movement as the photoexcited electrons can easily transfer from Bi_2_Te_3_ to Bi_2_Se_3_ and then from Bi_2_Se_3_ to Bi_2_S_3_, while the photogenerated holes can easily transfer from Bi_2_S_3_ to Bi_2_Se_3_ and then from Bi_2_Se_3_ to Bi_2_Te_3_.

The Bi_2_Te_3_/Bi_2_Se_3_/Bi_2_S_3_ cascade heterostructure was realized by a sequential deposition of Bi_2_S_3_, Bi_2_Se_3_, and Bi_2_Te_3_. The morphology by SEM (Figure 3d) and photograph (the inset) suggests that the centimeter‐scale Bi_2_Te_3_/Bi_2_Se_3_/Bi_2_S_3_ film is formed uniformly. The Raman spectrum of the Bi_2_Te_3_/Bi_2_Se_3_/Bi_2_S_3_ cascade heterostructure shows evident vibration modes from Bi_2_X_3_ with the $E_{g}^{2}$ (98.4 cm^−1^) and A_1g_ (140.7 cm^−1^) from Bi_2_Te_3_, the *E*_g_ (121.4 cm^−1^) and A_1g_ (164.8 cm^−1^) from Bi_2_Se_3_, and the A_g_ (184.2 and 236.5 cm^−1^) and B_1g_ (260.3 cm^−1^) from Bi_2_S_3_ as shown in Figure 3e. This result is consistent with the XPS in Figure S5 (Supporting Information), which further confirms the successful formation of the Bi_2_Te_3_/Bi_2_Se_3_/Bi_2_S_3_ cascade heterostructure.

**Figure 4:**
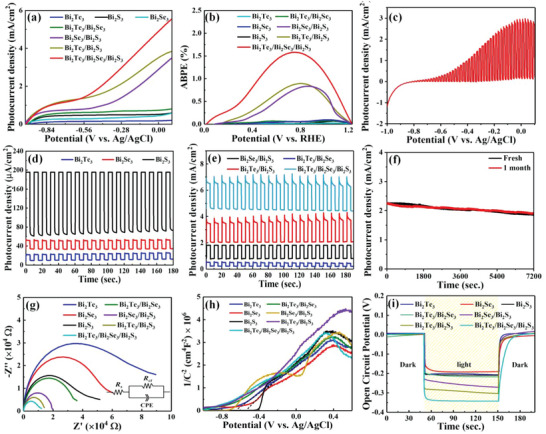
a) Cyclic linear sweep voltammetry (LSV) curves of different PEC‐type photodetectors based on Bi_2_X_3_ and heterostructures; b) ABPE result; c) Transient *I*–*V* measurement under the chopped light illumination; d) Photocurrent density of Bi_2_X_3_, and e) its type‐II heterostructure photodetectors under −0.1 V (relative to Ag/AgCl) bias voltage; f) Long cycle I–t curves of the Bi_2_Te_3_/Bi_2_Se_3_/Bi_2_S_3_ cascade heterostructure before and after one month under the illumination at −0.1 V; g) Nyquist plot and equivalent circuit model, h) Mott‐Schottky plots, and i) open circuit potential (OCP) decay curves of Bi_2_X_3_ and heterostructures.

Considering the well‐matched staggered bandgap (Figure 3c), the cascade heterostructures are expected to improve the PEC performance as the built‐in electric field at the multiple interfaces could promote the transmission of photoexcited electrons and holes. To validate the performance of these cascade heterostructures, we fabricated the PEC‐type photodetectors and carried out the photodetection measurements. Herein, photocurrent density (*I*_ph_) and photoresponsivity (*R*_ph_) are often used to quantitatively evaluate photodetection performance:^[^ ^23^ ^]^

(8) $$I_{\mathrm{ph}}=\left(I_{\mathrm{light}}-I_{\mathrm{dark}}\right)/S$$

(9) $$R_{\mathrm{ph}}=I_{\mathrm{ph}}/P_{\lambda }$$

where *I*_light_ and *I*_dark_ are the current responses under light illumination and dark states, respectively. In our experiment, the illumination area (*S*) and light power density (*P_*λ*_*) are 0.7 cm^2^ and 100 mWcm^−2^, respectively. It is evident from **Figure** **4**a that the photocurrent density of both pure Bi_2_X_3_ and the heterostructures increases with the bias voltage. It is worth noting that the photocurrent density of the Bi_2_Te_3_/Bi_2_Se_3_/Bi_2_S_3_ cascade heterostructure has a much higher value than those of pure Bi_2_X_3_ and their related type‐II heterostructures. To clearly evaluate the solar energy conversion efficiency, the applied bias photon‐to‐current efficiency ($\mathrm{ABPE}=\frac{I_{\mathrm{light}}\times \left(1.23-E_{\mathrm{RHE}}\right)}{P_{\lambda }}\times 100\%$) was calculated.^[^ ^4a^ ^]^ As shown in Figure 4b, the cascade heterostructure displays the largest ABPE among Bi_2_X_3_ and their related type‐II heterostructures. The maximum ABPE reaches 1.58% at the 0.75 V versus RHE equal to −0.1 V versus Ag/AgCl in our experiment, which is larger than those of BiVO_4_/CdS,^[^ ^24^ ^]^ ZnO/CuS,^[^ ^25^ ^]^ Fe_2_O_3_/NiFeOOH,^[^ ^26^ ^]^ CdS/TiO_2_,^[^ ^27^ ^]^ and Si/Au/TiO_2_.^[^ ^28^ ^]^ The ABPE improvement mainly comes from the high light absorption, efficient photogenerated electron‐hole separation, and fast carrier transport. As shown in Figure 4c from the *I*–*V* measurement under the chopped light illumination (100 mWcm^−2^), the PEC photodetector demonstrates a high photocurrent density without a bias or with a small bias. This suggests that this cascade heterostructure‐based photodetector can be used as a highly sensitive self‐powered photodetector as well as a low‐bias photodetector with a low on‐set potential.

We further demonstrate the transient photocurrent response of both pure Bi_2_X_3_ and heterostructures with a switching duration of 5 s as shown in Figure 4d,e under a low bias voltage (−0.1 V). Figure 4d demonstrates that the *I*_ph_ and *R*_ph_ values of Bi_2_S_3_ reach 131.5 µA cm^−2^ and 1315 µA W^−1^, which are higher than those of Bi_2_Se_3_ (*I*_ph_ ≈ 15.9 µA cm^−2^, *R*_ph_ ≈ 159 µA W^−1^) and Bi_2_Te_3_ (*I*_ph_ ≈ 12.3 µA cm^−2^, *R*_ph_ ≈ 123 µA W^−1^). This transient photocurrent response is determined by the carrier transportation and recombination rate of samples. Correspondingly, Figure 4e demonstrates the *I*_ph_ values of Bi_2_Te_3_/Bi_2_Se_3_ (301.2 µA cm^−2^), Bi_2_Se_3_/Bi_2_S_3_ (1047.6 µA cm^−2^), and Bi_2_Te_3_/Bi_2_S_3_ (1469.1 µA cm^−2^) are better than those of pure Bi_2_X_3_. It is evident from Figure 4e that the Bi_2_Te_3_/Bi_2_Se_3_/Bi_2_S_3_ cascade heterostructure demonstrates the highest *I*_ph_ and *R*_ph_ values (*I*_ph_ ≈ 2.2 mAcm^−2^, *R*_ph_ ≈ 22 mA W^−1^) among these pure bismuth chalcogenides and its type‐II heterostructures. These values are 178 times larger than that of Bi_2_Te_3_, 138 times larger than that of Bi_2_Se_3_, and 17 times larger than that of Bi_2_S_3_, respectively. Compared with the Bi_2_X_3_ heterostructure, the photoresponse of the cascade heterostructure is also greatly improved. The *I*_ph_ and *R*_ph_ values in the cascade heterostructure are 7.3, 2.1, and 1.5 times larger than those of the type‐II Bi_2_Te_3_/Bi_2_Se_3_, Bi_2_Se_3_/Bi_2_S_3_, and Bi_2_Te_3_/Bi_2_S_3_ heterostructures, respectively. The long‐term I‐t cyclic stability tests of Bi_2_Te_3_/Bi_2_Se_3_/Bi_2_S_3_ heterostructure are measured in Figure S6a–c (Supporting Information). The photocurrent signal exhibits a reversible behavior with the switchable light on and off, indicating excellent reproducibility and stability. Further long‐term photocurrent measurements show that the photocurrent density of the Bi_2_Te_3_/Bi_2_Se_3_/Bi_2_S_3_ was maintained almost constant ≈2.24 mA cm^−2^ before and after one month under the illumination at −0.1 V as shown in Figure 4f. The results demonstrate that the cascade heterostructure‐based photodetector also shows high stability. These improvements of the cascade heterostructure would be due to the rapid carrier transportation and slow electron‐hole pair recombination for the practical applications of PEC devices.

To further investigate the carrier dynamic process at the interface of heterogeneous structures, electrochemical impedance spectroscopy (EIS) was used as shown in Figure 4g. Generally, a smaller diameter in the EIS suggests a lower interfacial resistance, which can accelerate the charge transfer. In order to clearly understand the interface resistance, an equivalent circuit was constructed as shown in the inset in Figure 4g, where C*_PE_* and *R_s_* as well as *R_ct,_* represent the double layer capacitance, the electrolyte and charge transfer resistances, respectively. It is evident in Figure 4g that the semicircle diameter decreases in the order of Bi_2_Te_3_>Bi_2_Se_3_>Bi_2_S_3_>Bi_2_Te_3_/Bi_2_Se_3_>Bi_2_Se_3_/Bi_2_S_3_>Bi_2_Te_3_/Bi_2_S_3_>Bi_2_Te_3_/Bi_2_Se_3_/Bi_2_S_3_, which agrees well with LSV results. The results suggest that the cascade heterostructure shows a small interface resistance, due to the well‐matched band structure between Bi_2_X_3_ and ITO substrate. As such, the photoexcited electrons can transfer efficiently from Bi_2_Te_3_ to ITO substrate and generate a high photoresponse.

Furthermore, Mott‐Schottky curve is also used to analyze the performance of photodetectors. As shown in Figure 4h, it can be seen that the slope of the Mott‐Schottky curves is positive, which indicates that Bi_2_X_3_ and their heterostructures belong to n‐type semiconductors. In addition, the flat band potential (*V*_FB_) of the sample can be obtained by the tangent of the Mott‐Schottky curve with the X‐axis in Figure 4h. The *V*_FB_ of Bi_2_Se_3_, Bi_2_Te_3_, and Bi_2_S_3_ is calculated to be −0.53, −0.64, and −0.41 V (vs Ag/AgCl), respectively. Based on the *V*_FB_ measurement of these n‐type semiconductors,^[^ ^29^ ^]^ the band energy position of Bi_2_Te_3_/Bi_2_Se_3_/Bi_2_S_3_ in solution is also type‐II alignment, which agrees with the results in Figure 3c. Because of the more negative CB position of Bi_2_Te_3_, the electrons in the CB of Bi_2_Te_3_ will transfer to that of Bi_2_Se_3_ and then to that of Bi_2_S_3_.^[^ ^30^ ^]^ Furthermore, the inverse transfer direction of holes will greatly decrease the recombination of photogenerated carriers. These processes can also be found frequently in other cascade heterostructures such as ZnO/CdS/CdSe, ZnO/CdS/PbS, and MoS_2_/WS_2_/WSe_2_/Si.^[^ ^14^ ^]^ Furthermore, the *V*_FB_ of the Bi_2_Te_3_/Bi_2_Se_3_/Bi_2_S_3_ cascade heterostructure is also calculated to be −0.86 V, which is larger than those of Bi_2_Te_3_/Bi_2_Se_3_ (−0.70 V), Bi_2_Se_3_/Bi_2_S_3_ (−0.73 V), and Bi_2_Te_3_/Bi_2_S_3_ (−0.79 V) in Figure 4h. This suggests that the large band bending at the Bi_2_Te_3_/Bi_2_Se_3_/Bi_2_S_3_ cascade interfaces, benefiting for the charge separation due to the significantly sharp heterostructure interface, which is also found in ZnIn_2_S_4_/TiO_2_ and WO_3_/Bi_2_S_3_.^[^ ^30^ ^]^ The free carrier density (*N_d_*) can be obtained as follows:^[^ ^31^ ^]^

(10) $$N_{d}=\frac{2}{e\varepsilon_{0}\varepsilon_{r}}\left[\frac{dE}{d(\frac{1}{C^{2}})}\right]$$

where *e* = 1.6 × 10^−19^ C is the electron charge, and *ε*_0_ = 8.85×10^−14^ Fcm^−1^ is the vacuum permittivity; *ε_r_* is the relative permittivity of Bi_2_X_3_ (X = S, Te, Se).^[^ ^32^ ^]^ The calculated carrier density of Bi_2_Te_3_/Bi_2_Se_3_/Bi_2_S_3_ (≈4.9 × 10^24^ cm^−3^) is larger than those of Bi_2_Te_3_/Bi_2_Se_3_ (≈3.5 × 10^24^ cm^−3^), Bi_2_Se_3_/Bi_2_S_3_ (≈3.6 × 10^24^ cm^−3^), Bi_2_Te_3_/Bi_2_S_3_ (≈4.2 × 10^24^ cm^−3^), Bi_2_Se_3_ (≈3.1 × 10^24^ cm^−3^), Bi_2_Te_3_ (≈2.7 × 10^24^ cm^−3^), and Bi_2_S_3_ (≈3.4 × 10^24^ cm^−3^). The larger *N_d_* values are easier to raise the Fermi‐level closer to their conduction band position and then decrease the *V*_FB_, indicating a faster charge transfer.

In addition, the OCP measurements in Figure 4i also verify the n‐type semiconductors according to the low OCP under the light state.^[^ ^33^ ^]^ Here, the photovoltage (*V*_ph_) is defined as OCP_dark_−OCP_light_ and the *V*_ph_ is caused by the Fermi‐level pinning effect. In our experiment, the cascade heterostructure shows the highest *V*_ph_ value (≈0.35 eV) than those of Bi_2_X_3_ and its type‐II heterostructures. This is because heterostructure formation could eliminate the Fermi‐level pinning induced by the trap state,^[^ ^34^ ^]^ resulting in a high *V*_ph_ and a large band bending between the photoanode and electrolyte interface. The OCP measurement agrees well with the Mott‐Schottky and EIS results as the sharp band bending in the cascade heterostructure can effectively suppress the surface carrier recombination and promote the surface charge transport ability.^[^ ^35^ ^]^

**Figure 5:**
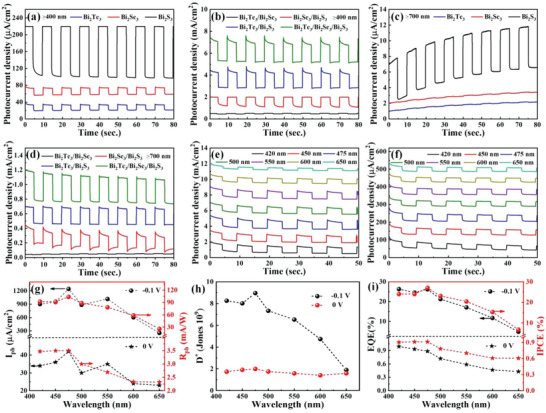
Transient photocurrent response curves of a) Bi_2_X_3_ and b) heterostructures in visible region; Transient photocurrent response curves of c) Bi_2_X_3_ and d) heterostructures in the wavelength band ≥700 nm; The photoresponse of the cascade heterostructure under the bias of e) −0.1 and f) 0 V; g) *I*_ph_ and *R*_ph_ values in visible region under the bias voltage of −0.1 and 0 V; h) D* and i) EQE and IPCE values of the Bi_2_Te_3_/Bi_2_Se_3_/Bi_2_S_3_ cascade heterostructure at the bias voltage of −0.1 and 0 V.

To investigate the effect of light absorption range on the photodetection ability, the *I*–*t* measurement was conducted under the wavelengths ≥400 nm and ≥700 nm by adding band‐pass optical filters. Similar to LSV results, the Bi_2_Te_3_/Bi_2_Se_3_/Bi_2_S_3_ cascade heterostructure also shows the highest *I*_ph_ (1.77 mA cm^−2^ and 373 µA cm^−2^) and *R*_ph_ (29.5 and 18.65 mA W^−1^) than those of Bi_2_X_3_ and its type‐II heterostructures under the wavelengths ≥400 nm and ≥700 nm as shown in **Figure** **5**a‐d, respectively. The *I*_ph_ and *R*_ph_ values of Bi_2_X_3_ and heterostructures are summarized in Table S1 (Supporting Information). In contrast, the *I*_ph_ at the wavelength ≥400 nm is much larger than that of ≥700 nm due to the efficient light absorption in visible region. Additionally, the efficient charge separation and transfer in the cascade heterostructure also have a synergy effect to produce high photoresponse.

To deeply explore the visible‐light‐driven photodetector performance, the photoresponse of the Bi_2_Te_3_/Bi_2_Se_3_/Bi_2_S_3_ cascade heterostructure at −0.1 V was investigated under different visible light wavelengths as shown in Figure 5e. The cascade heterostructure‐photodetector keeps high *I*_ph_ values in Table S2 (Supporting Information). Especially, the *I*_ph_ reaches the maximum value ≈1243 µA cm^−2^ at the wavelength of 475 nm, which may be due to the high light‐absorption at the special wavelength.^[^ ^5^ ^]^ To estimate the sensitivity performance of the cascade heterostructure, the *R*_ph_ as a function of the wavelength is displayed in Figure 5g. The *R*_ph_ increases from 92 to 103 mAW^−1^ with the wavelength increased from 420 to 475 nm, and then it gradually decreases to 25.76 mA W^−1^. The *I*_ph_ and *R*_ph_ values are much larger than those of previously reported PEC photodetectors as shown in **Table** **1**. It is evident that the *R*_ph_ value of the cascade heterostructure is approximately 10^3^ times larger than those of 2D GeSe and SnS^[^ ^1^, ^36^ ^]^ and ≈10^2^ times larger than those of 2D SnS/SnSe_2_ and Te@Se heterostructures.^[^ ^37^ ^]^ This is mainly determined by the strong light absorption ability, well‐matched band alignment, and specific atomic‐level interfacial contact in the Bi_2_Te_3_/Bi_2_Se_3_/Bi_2_S_3_ cascade heterostructure. The superior photoresponse performance of the cascade heterostructure suggests a great commercial potential in the visible‐light‐driven PEC photodetectors.

Further exploration of the photodetection behavior in the visible region without an applied voltage was also investigated as shown in Figure 5f and the relationship between the *I*_ph_ (*R*_ph_) and wavelengths is a similar to those in Figure 5e. The cascade heterostructure‐based photodetector shows an excellent self‐powered performance with the maximum *I*_ph_ value of 42 µAcm^−2^ and *R*_ph_ value of 3.5 mAW^−1^ at 475 nm, which are better than those of previously reported 2D materials‐based PEC photodetector as shown in Table S3 (Supporting Information). On the one hand, the 2D Bi_2_X_3_ have a strong light absorption efficiency beyond 10^4^ cm^−1^ even in the infrared region due to the narrow bandgap, which can produce more photogenerated carriers compared with those of 2D nanosheets in Table S3 (Supporting Information). On the other hand, the Bi_2_Te_3_/Bi_2_Se_3_/Bi_2_S_3_ cascade heterostructure generates strong built‐in electric fields at the multiple interfaces and then promotes the photoexcited electron‐hole transportation, resulting in a high self‐powered capacity. This self‐powered photodetector meets the practical application requirement in various harsh and complicated environments with low‐energy consumption, and light weight.

Apart from *R*_ph_, specific detectivity (D*) is another key parameter to estimate the photo‐responsiveness of photodetectors. The D* values are calculated by Equation 11 and summarized in Figure 5h and Table S4 (Supporting Information).

(11) $$D^{*}=R_{\mathrm{ph}}\times S^{1/2}/{\left(2q\times I_{\mathrm{dark}}\right)}^{1/2}$$

It is evident that the cascade heterostructure also shows the strongest detectivity (8.96 × 10^9^ and 1.99 × 10^9^ Jones for −0.1 V and 0 V at 475 nm) than those of other wavelengths. Additionally, the D* values of the cascade heterostructure‐based photodetectors are an order of magnitude larger than those of PEC‐type photodetectors such as Bi,^[^ ^44^ ^]^ Te,^[^ ^45^ ^]^ BP,^[^ ^46^ ^]^ SnS,^[^ ^36a^ ^]^ and Te@Bi.^[^ ^47^ ^]^ High crystallinity and efficient light absorption as well as fast interfacial charge transportation of cascade heterostructure lead to a low dark current and a high detectivity.

To quantitatively evaluate the efficiency of the cascade heterostructure based photodetectors, the external quantum efficiency (EQE) and incident photon‐to‐current efficiency (IPCE) are calculated based on the incident photon as a function of the wavelength as follows:

(12) $$\mathrm{EQE}=h\times c\times R_{ph}/\left(q\times \lambda \right)$$

(13) $$\mathrm{IPCE}=\frac{1240}{\lambda }\times \frac{I_{\mathrm{light}}-I_{\mathrm{dark}}}{P_{\lambda }}\times 100\%$$

Here, *q* is 1.6 × 10^−19^ C; *h* is 6.63 × 10^−34^ J s; *c* is 3 × 10^8^ m s^−1^; *λ* is the incident wavelength. The calculated EQE and IPCE are summarized in Figure 5i and Table S4 (Supporting Information). It is clear that the cascade heterostructure photodetector displays a broad and high EQE and IPCE values in the visible region. Significantly, the cascade heterostructure exhibits the highest EQE (26.17% and 0.88% for −0.1 and 0 V) and IPCE (27.04% and 0.91% for −0.1 V and 0 V) at the wavelength of 475 nm in consistent with the wavelength‐dependent photoresponse results in Figure 5g. These results further suggest that the cascade heterostructure can effectively accelerate the carrier separation and transportation under a small bias voltage with a large junction area.

**Figure 6:**
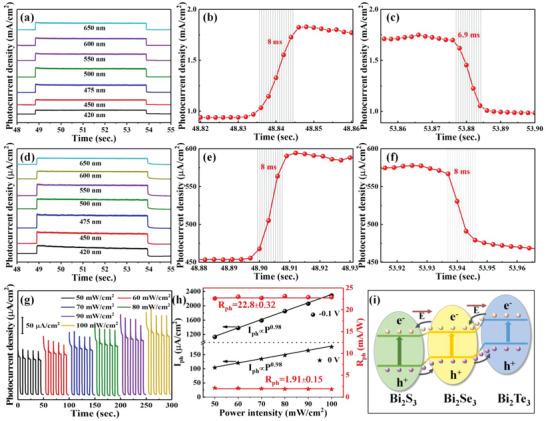
a) *t*_res_/*t*_rec_ measurement at different incident light wavelengths, b) *t*_res_ and c) *t*_rec_ at 475 nm under the bias voltage of −0.1 V; d) *t*_res_/*t*_rec_ measurement at different incident light wavelengths, e) *t*_res_ and f) *t*_rec_ at 475 nm under the bias voltage of 0 V; g) Photocurrent density of the Bi_2_Te_3_/Bi_2_Se_3_/Bi_2_S_3_ photodetector at ‐0.1 V, and h) the extracted *I*_ph_ and *R*_ph_ values under different power intensities at −0.1 and 0 V; i) Working mechanism of self‐powered photodetector.

To evaluate the sensitivity of the photodetector, the response time (*t*_res_, response from 10% to 90%) and recovery time (*t*_rec_, recombination from 90% to 10%) are measured under the single wavelength as shown in **Figure** **6**a. Interestingly, the *t*_res_ and *t*_rec_ are on a millisecond scale, which is almost not influenced by the incident wavelength. Figure 6b,c show the typical *t*_res_ (8 ms) and *t*_rec_ (6.9 ms) of the Bi_2_Te_3_/Bi_2_Se_3_/Bi_2_S_3_ cascade heterostructure at 475 nm. Even without a voltage bias voltage, the cascade heterostructure still keeps the fast response of *t*_res_ (8 ms) and *t*_rec_ (8 ms) at different incident wavelengths as shown in Figure 6d–f. The *t*_res_ value is comparable to the *t*_rec_ value, suggesting that few defects and trap centers are involved in carrier separation and recombination. The rapid photoresponse characteristics in the cascade heterostructure are far superior to those of 2D materials‐based photodetectors. For instance, the response time in the cascade heterostructure is an order of magnitude faster than previously reported Bi_2_S_3_‐based photodetector (*t*_res_ ≈100 ms and *t*_rec_ ≈100 ms).^[^ ^48^ ^]^ On the one hand, the rapid response could result from the internal electric field of the multi‐staggered band alignment that induces a fast charge transfer and separation efficiently. On the other hand, the rapid response could result in a strong redox reaction, which unambiguously promotes the PEC process, leading to a high photocurrent density.

To further evaluate the light sensitive properties of the photodetector, the incident light power‐dependent photoresponse is investigated with the power intensity from 50 to 100 mW cm^−2^ at −0.1 V as shown in Figure 6g. The extracted *I*_ph_ from Figure 6g increases from 1130 to 2390 µA cm^−2^ at −0.1 V. Similarly, without a bias voltage, the *I*_ph_ also increases proportionally from 104 to 178 µA cm^−2^ as shown in Figure S7 (Supporting Information). Furthermore, the *I*_ph_ increase almost linearly with the increment of power intensity as shown in Figure 6h, which can be fitted by *I*_ph_∝*P* ^0.98^ for both with and without a bias voltage. Based on the photocurrent generation principle, *I*_ph_ would be linearly dependent on the incident power density under ideal conditions for the photoconductive detector.^[^ ^49^ ^]^ As such, the linear power dependence suggests the high crystallinity of the Bi_2_Te_3_/Bi_2_Se_3_/Bi_2_S_3_ cascade heterostructure with relatively low defects and traps.^[^ ^50^ ^]^ Apart from the *I*_ph_ values, the detailed *R*_ph_ values are also calculated and summarized into Table S5 (Supporting Information). The extracted *R*_ph_ values keep 22.8 ± 0.32 and 1.91 ± 0.15 mAW^−1^ at −0.1 and 0 V as shown in Figure 6h, suggesting a high PEC detection stability even under a weak visible light. It is worth pointing out that the *R*_ph_ values in the cascade heterostructure are ≈10^2^–10^3^ times larger than those of GeSe and Te nanosheets based self‐powered photodetector.^[^ ^1^, ^45^ ^]^ The large‐area junction interface and few trap states are responsible for this improvement. The self‐powered characteristics are well explained based on the energy‐band structure in Figure 6i. The built‐in electric field at the semiconductor interface can ensure that photodetector works well even without a bias voltage. The charge transfer in the heterstrucrure is mainly determined by both Fermi level and the energy difference (Δ*E*_cv_) between CB of semiconductor I and VB of semiconductor II.^[^ ^51^ ^]^ According to the band arrangement of the Bi_2_Te_3_/Bi_2_Se_3_/Bi_2_S_3_ heterostructure, a smaller work function (higher Fermi level) of semiconductor Bi_2_S_3_ than those of Bi_2_Se_3_ and Bi_2_Te_3_, which is characterized by ultraviolet photoelectron spectroscopy measurements as shown in Figure S8 (Supporting Information). Furthermore, the energy difference (Δ*E*_cv_ ≈ 0.73 eV) between CB of Bi_2_S_3_ and VB of Bi_2_Se_3_ is far larger than that of Δ*E*_c_ (0.07 eV). Similarly, the Δ*E*_cv_ (0.5 eV) between CB of Bi_2_Se_3_ and VB of Bi_2_Te_3_ is larger than that of Δ*E*_c_ (0.32 eV) as shown in Figure 3b. The built‐in electric field at the Bi_2_Te_3_/Bi_2_Se_3_/Bi_2_S_3_ heterostructure interface favors the type‐II charge transfer process. Furthermore, the enhanced photocurrent in the heterostructure is solid evidence to verify the type‐II instead of direct Z‐scheme heterostructure in Figure S9 (Supporting Information), which is consistent with the ultraviolet photoelectron spectroscopy measurement results. The photoresponse of Bi_2_S_3_/Bi_2_Se_3_ and Bi_2_Se_3_/Bi_2_S_3_ (Bi_2_Te_3_/Bi_2_Se_3_ and Bi_2_Se_3_/Bi_2_Te_3_) heterostructures was also measured as shown in Figure S10a,b (Supporting Information). Compared with Bi_2_S_3_/Bi_2_Se_3_/ITO heterostructure, much more photogenerated electrons of Bi_2_Se_3_/Bi_2_S_3_/ITO heterostructure are collected at ITO substrate and then generate a higher photocurrent as shown in Figure S11 (Supporting Information). This is in consistent with our experimental results as shown in Figure S10a (Supporting Information). Furthermore, the photocurrent of Bi_2_S_3_ is larger than that of Bi_2_S_3_/Bi_2_Se_3_/ITO heterostructure. This is because the photogenerated electrons of Bi_2_Se_3_ quickly transfer toward Bi_2_S_3_ and then participate in water reduction reaction as shown in Figure S11d (Supporting Information). The results further confirm that the Bi_2_Se_3_/Bi_2_S_3_ belongs to type‐II heterostructure instead of Z‐scheme heterostructure as shown in Figure S11 (Supporting Information). Similarly, the photocurrent measurement in Figure S10b (Supporting Information) also demonstrates that the Bi_2_Te_3_/Bi_2_Se_3_ heterostructure also belongs to type‐II heterostructure. Under the light illumination, the photoexcited electron‐hole pairs are separated by the internal electric field. In details, the photoinduced electrons transmit from valence band (VB) to conduction band (CB) of the Bi_2_X_3_ semiconductors and the electrons in the CB of Bi_2_Te_3_ would go into the CB of the Bi_2_Se_3_ and then flow into Bi_2_S_3_ as shown in Figure 6i.

**Figure 7:**
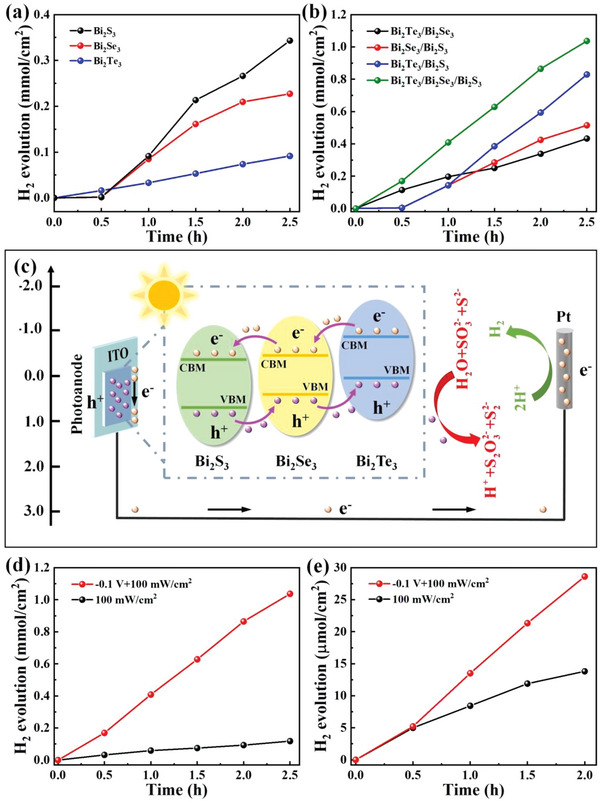
PEC hydrogen production of a) Bi_2_X_3_ and b) heterojunctions under the visible light illumination at the bias voltage of −0.1 V; c) PEC hydrogen production mechanism of the Bi_2_Te_3_/Bi_2_Se_3_/Bi_2_S_3_ cascade heterostructure; PEC hydrogen production of Bi_2_Te_3_/Bi_2_Se_3_/Bi_2_S_3_ cascade heterostructure under 100 mWcm^−2^ in d) sacrificial agent, and e) pure water.

Different from other photodetectors, the PEC photodetectors can work in electrolytes and the electrolytes as ion channels to complete the current loop. Under the light illumination, this PEC‐type photodetector not only can collect the photogenerated electrons to produce electric signal, but also can produce H_2_ due to the photogenerated electrons undergoing the redox reactions. A vacuum gas circulation system combined with a gas chromatograph was employed to detect the amount of H_2_ production (see Experimental Section for details). Under the bias voltage of −0.1 V, the H_2_ productions are 0.23, 0.09, and 0.34 mmol cm^−2^ for Bi_2_Se_3_, Bi_2_Te_3,_ and Bi_2_S_3_ within 2.5 h as shown in **Figure** **7**a. The results suggest that the Bi_2_X_3_ films show strong activity under a small bias voltage as shown in Table S6 (Supporting Information). Especially, compared with previously reported Bi_2_S_3_ nanowires,^[^ ^52^ ^]^ our chemical vapor deposited Bi_2_S_3_ film shows the almost two‐fold increase in the PEC water splitting performance as shown in Table S6 (Supporting Information). This is mainly due to the reason that the atomic‐level interfacial contact between the centimeter‐scale Bi_2_X_3_ film and ITO substrate can accelerate the charge transport to counter electrode for reduction reaction.

To further improve the water splitting performance, the Bi_2_X_3_ heterostructures are investigated on the PEC hydrogen production under the same measurement conditions in Figure 7b. The H_2_ productions of Bi_2_Te_3_/Bi_2_Se_3_, Bi_2_Se_3_/Bi_2_S_3_, and Bi_2_Te_3_/Bi_2_S_3_ films are 0.43, 0.51, and 0.83 mmol cm^−2^ within 2.5 h which are 4.78, 5.67, and 9.22 times larger than that of Bi_2_Te_3_ under the same conditions. Interesting, the H_2_ production rate of Bi_2_Te_3_/Bi_2_S_3_ is also superior to those of TiO_2_/Bi_2_Se_3_, Bi_2_Te_3_/V_0.04_‐Sb_2_Te_3_, Bi_2_S_3_/BiVO_4_, and Bi_2_S_3_@TiO_2_ heterostructures^[^ ^10^, ^11^, ^53^ ^]^ as shown in Table S6 (Supporting Information). This improvement in the Bi_2_X_3_ heterostructures is mainly attributed to several merits. I) Two‐step vapor deposited van der Waals heterostructures can effectively avoid the alloy and defect‐center formation. II) The similar crystal structure among 2D Bi_2_X_3_ materials is beneficial for forming a good‐contact and a large‐area heterostructure interface. III) The conducting surface state facilitates the charge transfer at the large‐area interface. IV) The formed type‐II heterostructure can effectively separate electron‐hole pairs and participate the redox reaction. As a representative Bi_2_X_3_ heterostructure, the charge transfer process of Bi_2_Se_3_/Bi_2_S_3_ can be described as following. Under the visible light illumination, the photogenerated electrons transmit from VB to CB of Bi_2_Se_3_ and Bi_2_S_3_ semiconductors and the electrons in the Bi_2_Se_3_ would go into the CB of the Bi_2_S_3_. Much more accumulated electrons in the Bi_2_S_3_ would transit to the counter electrode and generate a higher H_2_ production rate than those of pure Bi_2_X_3_. Similarly, the interfacial charge transfer also improves the H_2_ production rates in the Bi_2_Te_3_/Bi_2_Se_3_ and Bi_2_Te_3_/Bi_2_S_3_ heterostructures.

Considering the interfacial carrier transportation, the Bi_2_Te_3_/Bi_2_Se_3_/Bi_2_S_3_ heterostructure with a multi‐staggered bandgap and multiple interfaces was designed and the H_2_ production can reach 1.04 mmol cm^−2^, which is 4.5, 11.6, and 3.06 times larger than those of Bi_2_Se_3_, Bi_2_Te_3,_ and Bi_2_S_3_, respectively. Similarly, the H_2_ production performance of Bi_2_Te_3_/Bi_2_Se_3_/Bi_2_S_3_ is also far larger than those of Bi_2_X_3_‐based heterostructures as shown in Table S6 (Supporting Information). Especially for a low bias voltage, the H_2_ production in the cascade heterostructure is 17.5 times larger than the Bi_2_S_3_‐BiOBr/TiO_2_ heterostructures.^[^ ^54^ ^]^ The highest H_2_ production rate in the cascade heterostructure is realized among the 2D Bi_2_X_3_ materials and related heterostructures, which mainly come from the efficient charge transport and proper band alignment as shown in Figure 7c. The electron‐hole pairs are separated in the cascade heterostructure based photoanode under the simulated sunlight illumination. The well‐matched band energy edge at the Bi_2_Te_3_/Bi_2_Se_3_/Bi_2_S_3_ cascade heterostructure interfaces provides an efficient carrier highway to deliver electrons to counterpart electrode and reduce the recombination of photogenerated carriers. Under a small applied voltage, the carrier transport rate was also greatly improved due to the external electric field. Meanwhile, the holes participate in the oxidation reaction with the sacrificial agent (Equations (14), (15), (16), (17)) and the corresponding schematic diagram of the photocatalytic mechanism is also displayed in Figure 7c. The $S_{2}^{2-}$ production can be efficiently inhibited by the mixing ${\mathrm{SO}}_{3}^{2-}$ ions and then produce $S_{2}O_{3}^{2-}$ ions. In the chemical reaction process, the holes are consumed and further slow the carrier recombination, resulting in a high PEC performance.

(14) $${\mathrm{SO}}_{3}^{2-}+H_{2}{O+2h}_{\mathrm{VB}}^{+}\to {\mathrm{SO}}_{4}^{2-}+2H^{+}$$

(15) $$2S^{2-}{+2h}_{\mathrm{VB}}^{+}\to S_{2}^{2-}$$

(16) $$S_{2}^{2-}{+\mathrm{SO}}_{3}^{2-}\to S_{2}O_{3}^{2-}+S^{2-}$$

(17) $${\mathrm{SO}}_{3}^{2-}+S^{2-}{+2h}_{\mathrm{VB}}^{+}\to S_{2}O_{3}^{2-}$$

In order to deeply understand the relationship between PEC and photocatalytic H_2_ production, the H_2_ amount of the Bi_2_Te_3_/Bi_2_Se_3_/Bi_2_S_3_ cascade heterostructure at the bias voltage of −0.1 and 0 V under 100 mW cm^−2^ was measured in Figure 7d. The result shows that the H_2_ production can reach 0.12 mmol cm^−2^ within 2.5 h even under 0 V. Significantly, H_2_ production can greatly be improved to 1.04 mmol cm^−2^ under a small external bias voltage of −0.1 V due to the synergistic effect. However, hydrogen evolution takes place at the high‐cost consumption of electron sacrificial agents in the PEC processing. In our experiment, the PEC and photocatalytic H_2_ production of the Bi_2_Te_3_/Bi_2_Se_3_/Bi_2_S_3_ cascade heterostructure in pure water was further tested as shown in Figure 7e. The results show that the Bi_2_Te_3_/Bi_2_Se_3_/Bi_2_S_3_ cascade heterostructure can produce 13.81 µmol cm^−2^ of photocatalytic H_2_ production and 28.64 µmol cm^−2^ of PEC hydrogen production within 2 h, respectively. This is mainly from water reduction reaction, which can be described as 2H_2_O + 2e^‐^= 2OH^‐^+H_2_. This result shows that the Bi_2_Te_3_/Bi_2_Se_3_/Bi_2_S_3_ cascade heterostructure can realize high‐activity photocatalytic H_2_ production and PECH_2_ production even without using sacrificial agent.

## 3 Conclusion

The Bi_2_Te_3_/Bi_2_Se_3_/Bi_2_S_3_ cascade heterostructure with a well‐matched band alignment was optimized and prepared by a vapor phase deposition method. The cascade heterostructure‐based PEC photodetector exhibits a fast response at a millisecond level, a high photoresponsivity in 10^2^ mAW^−1^ scale, and a high detectivity beyond 10^9^ Jones under a small bias of −0.1 V. Furthermore, the Bi_2_Te_3_/Bi_2_Se_3_/Bi_2_S_3_ photodetector also demonstrates a superior self‐powered capability, displaying a broad photoresponse in the visible region. The excellent photodetection performance in the Bi_2_Te_3_/Bi_2_Se_3_/Bi_2_S_3_ cascade heterostructure is mainly attributed to the efficient charge transfer at the multiple interfaces, which is characterized by EIS, OCP, and Mott‐Schottky measurements. Benefiting from their proper band position, efficient light harvesting ability, and high charge transport efficiency, the cascade heterostructure also shows a superior photocatalytic activity and the H_2_ generation rate can reach 0.416 mmol cm^−2^h^−1^ and 14.320 µmol cm^−2^ h^−1^ with or without the sacrificial agent, respectively. The incorporation of merits of cascade heterostructure is very promising for PEC photodetector and water splitting applications.

## 4 Experimental Section

### Materials

Bismuth(III) telluride (Bi_2_Te_3_, 99.999%), bismuth(III) selenide ((Bi_2_Te_3_, 99.999%), and bismuth(III) oxide (Bi_2_O_3_, 99%) were purchased from Alfa. Sulfur (S, 99.998%) powder was purchased from Sigma‐Aldrich. Sodium sulfide (Na_2_S, >98%) and sodium sulfite (Na_2_SO_3_, 98.5%) were purchased from Acros.

### Synthesis of Bi_2_X_3_ Materials

In the experiment, 2D uniform Bi_2_Se_3_ films were deposited onto ITO substrate by using a PVD method. Before the growth process, Ar gas flow (200 sccm) was filled the furnace to drive away air and the furnace was pumped down to 100 pa. During the growth process, 5 mg Bi_2_Se_3_ powder was heated to 500 °C within 20 minutes and kept for 5 min and the vapor was carried onto the ITO substrate by 50 sccm Ar gas. The Bi_2_Se_3_ nanosheet deposits onto the ITO substrate at the distance of 10 cm from the source, where the temperature is 350 °C. Due to the similar physical and chemical properties of Bi_2_Se_3_ and Bi_2_Te_3_, the same synthesis parameters were also used to grow Bi_2_Te_3_ by the PVD method. Compared with the PVD method, the Bi_2_S_3_ film was synthesized by a CVD method. Bi_2_O_3_(5 mg) and 100 mg S powders were selected as precursors and located at 650 and 180 °C in a tube furnace with two independent temperature zones, respectively. Two independent temperature zones were heated to 650 and 180 °C within 25 minutes and the temperature was kept for 5 minutes to grow Bi_2_S_3_ film. Ar gas flow was maintained at 50  ccm and the inside pressure was controlled at 300 pa. The ITO substrate is put ≈15 cm away from the sources to deposit Bi_2_S_3_ nanosheets, where the substrate temperature is 400 °C. Bi_2_Te_3_/Bi_2_Se_3_, Bi_2_Se_3_/Bi_2_S_3_, and Bi_2_Te_3_/Bi_2_S_3_ were synthesized by a two‐step vapor phase deposition method according to the above‐mentioned growth process. To prepare Bi_2_Te_3_/Bi_2_Se_3_/Bi_2_S_3_ cascade heterostructure, Bi_2_S_3_ film was first deposited onto the ITO substrate. Subsequently, Bi_2_S_3_/ITO acts as a new substrate to continually grow Bi_2_Se_3_ to form Bi_2_Se_3_/Bi_2_S_3_/ITO heterostructure. Finally, Bi_2_Te_3_ film was deposited onto Bi_2_Se_3_/Bi_2_S_3_/ITO heterostructure to construct the Bi_2_Te_3_/Bi_2_Se_3_/Bi_2_S_3_ cascade heterostructure.

### Characterization

SmartRaman confocal‐micro‐Raman module (Institute of Semiconductors, Chinese Academy of Sciences) with the laser excited at 532 nm was used to characterize the vibration modes of Bi_2_X_3_ films. X‐Ray photoelectron spectroscopy (XPS, Thermo Fisher, ESCALAB Xi+) was used to confirm the bonding configuration and electronic structure of the samples. Scanning electron microscopy (SEM, Thermo Fisher, Apreo S) was used to observe the morphology. The semiconductor crystal phase was confirmed by X‐ray diffraction spectroscopy (XRD, Bruker, D8 Advance). UV–vis absorption spectroscopy (R1, Ideaoptics) was employed to confirm the light absorption.

### PEC Measurements

The photodetection performance of the PEC photodetectors was characterized by a traditional three‐electrode PEC system in a quartz reaction cell filled with a pH = 11 solution electrolyte (0.1 molL^−1^ Na_2_S and 0.02 molL^−1^ Na_2_SO_3_). *I*–*V* curve was measured by the cyclic linear sweep voltammetry from −1 to 0.1 V at a scan rate of 10 mVs^−1^. The photoresponse was investigated under the irradiation of simulated light ranging from 400 to 780 nm. The transient photocurrent response was measured with a switching duration of 5 s. The PEC photodetectors were illuminated under the visible light wavelength of 420, 450, 475, 500, 550, 600, and 650 nm. The light power intensities (50, 60, 70, 80, 90, and 100 mWcm^−2^) were used to investigate the relationship between the photoresponse and the pump intensity. The EIS spectra were measured in the frequency range of 0.01 Hz to 100 kHz under the conditions of the dark environment and open circuit voltage.

### Hydrogen Evolution Measurement

All glass automatic on‐line trace gas analysis system (Labsolar‐6A, Beijing Perfectlight) was used to collect and detect the hydrogen (H_2_) production under the irradiation of 100 mWcm^−2^. The hydrogen produced was then quantitatively analyzed using a gas chromatograph (GC9790Plus, FULI INSTRUMENTS). The mixed solution of 0.1 molL^−1^ Na_2_S and 0.02 molL^−1^ Na_2_SO_3_ was selected as the sacrificial agent.

## Conflict of Interest

The authors declare no conflict of interest.

## Supporting information

Supporting Information

## Data Availability

The data that support the findings of this study are available from the corresponding author upon reasonable request.
