# Supplementary material for: Bi2Te3/Bi2Se3/Bi2S3 Cascade Heterostructure for Fast‐Response and High‐Photoresponsivity Photodetector and High‐Efficiency Water Splitting with a Small Bias Voltage
Source: Adv Sci (Weinh). 2022 Dec 27;10(6):2205460. doi: 10.1002/advs.202205460 (PMC9951346; doi:10.1002/advs.202205460)
Supplement: Supplementary file 1 — Supporting Information [file ADVS-10-2205460-s001.pdf]

ADVANCED SCIENCE
Open Access

Supporting Information

for *Adv. Sci.*, DOI 10.1002/advs.202205460

$Bi_2Te_3/Bi_2Se_3/Bi_2S_3$ Cascade Heterostructure for Fast-Response and High-Photoresponsivity Photodetector and High-Efficiency Water Splitting with a Small Bias Voltage

Chunhui Lu, Mingwei Luo, Wen Dong, Yanqing Ge, Taotao Han, Yuqi Liu, Xinyi Xue, Nan Ma, Yuanyuan Huang, Yixuan Zhou* and Xinlong Xu**

Supporting Information

$Bi_2Te_3/Bi_2Se_3/Bi_2S_3$ cascade heterostructure for fast-response and high-photoresponsivity photodetector and high-efficiency water splitting with a small bias voltage

Chunhui Lu †, Mingwei Luo †, Wen Dong, Yanqing Ge, Taotao Han, Yuqi Liu, Xinyi Xue, Nan Ma, Yuanyuan Huang, Yixuan Zhou*, Xinlong Xu**

1. Optical and scanning electron microscopy (SEM) characterization

From the photographs of Bi_2X_3 (X=Se, Te, and S) and their type-II heterostructures in **Figure S1(a-f)**, the centimeter-scale nanofilms are synthesized by a vapor deposition method. To clearly observe the surface morphology, the SEM was employed and the results show that the Bi_2X_3 nanosheets are deposited and form large-area continuous films on the ITO substrates.

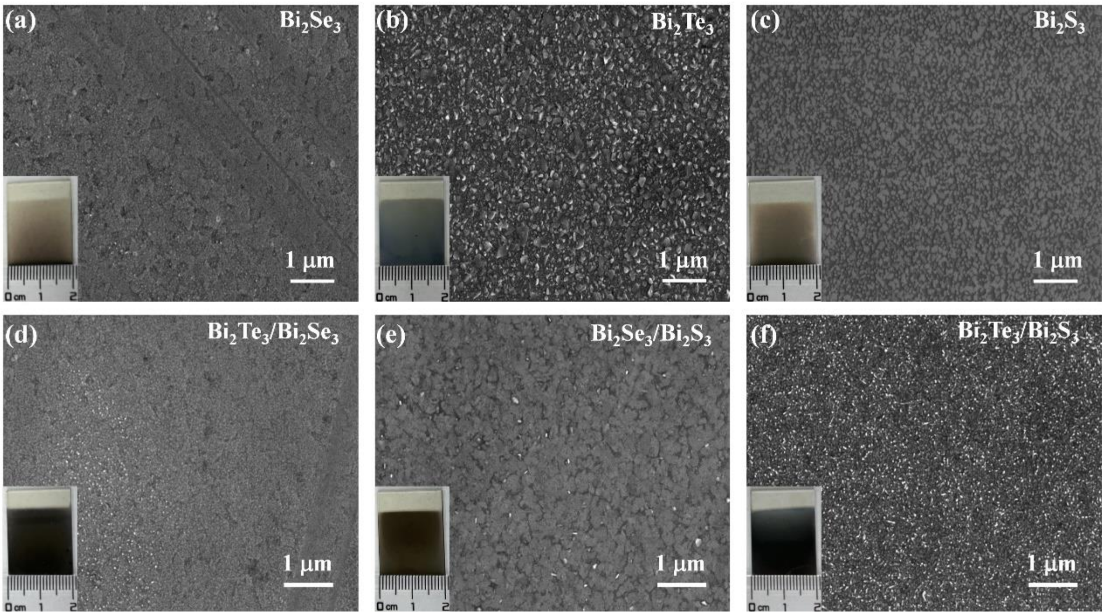

Figure S1. (a-f) Photographs and SEM images of Bi_2X_3 (X=Se, Te, and S) and their type-II heterostructures.

2. X-ray diffraction spectroscopy measurement

X-ray diffraction spectroscopy (XRD, Bruker, D8 Advance) was employed to confirm the

crystal phase of the prepared films. As shown in **Figure S2(a)**, peaks at 23.54, 28.07, 40.95, and 47.56° correspond to the (1 2 1), (0 0 9), (1 0 10), and (0 0 15) diffraction peaks of hexagonal phase Bi_2Se_3 (JCPDS No. 33-0214), consistent with the previous report.[1] As shown in **Figure S2(b)**, the peak at 19.14° corresponds to the (0 0 6) diffraction peak of hexagonal phase Bi_2Te_3 (JCPDS No. 15-0863).[2] Meanwhile, peaks at 16.22, 18.27, 22.37, 25.54, 29.20, 32.65, and 39.65° correspond to the (2 0 0), (1 2 0), (3 1 0), (2 1 1), (3 0 1), (0 4 1), and (4 3 0) diffraction peaks of orthorhombic phase Bi_2S_3 (JCPDS No. 17-0320),[3] as shown in **Figure S2(c)**.

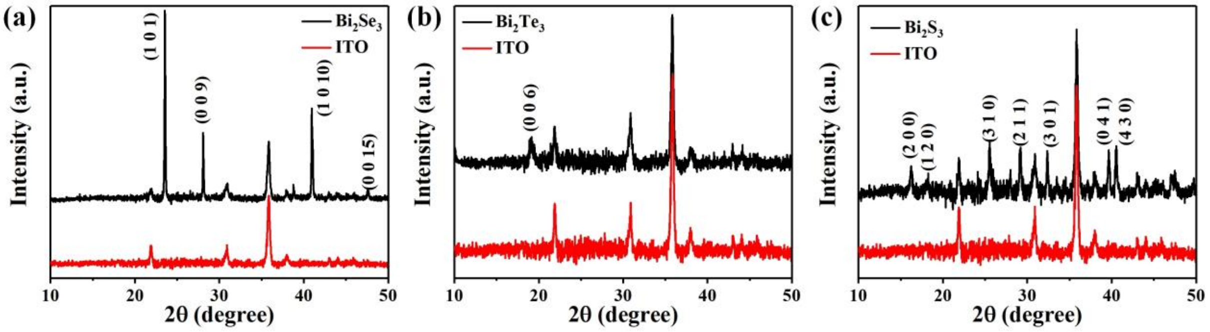

Figure S2. XRD patterns of (a) Bi_2Se_3, (b) Bi_2Te_3, and (c) Bi_2S_3 films.

3. X-ray photoelectron spectroscopy (XPS) measurement

The Te and Se core levels in the Bi_2Te_3/Bi_2Se_3 heterostructure, Se and S core levels in the Bi_2Se_3/Bi_2S_3 heterostructure, and Te and S core levels in the Bi_2Te_3/Bi_2S_3 heterostructure are fitted and shown in **Figure S3(a-b)**, **(c-d)**, and **(e-f)**, respectively. These core levels in the heterostructures have a shift toward higher or lower binding energy compared with those of pure Bi_2Te_3, Bi_2Se_3, and Bi_2S_3 as shown in Figure 1 in the main text, indicating the successful heterostructure formation and interfacial carrier redistribution.

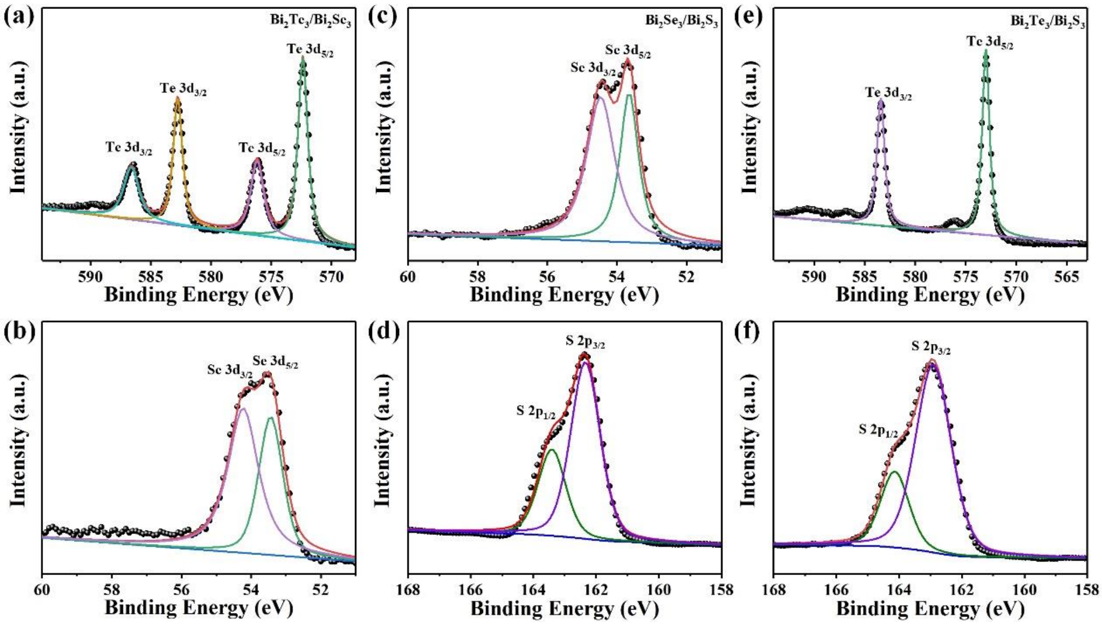

Figure S3. XPS spectra of (a) Te 3d core level and (b) Se 3d core level of the Bi_2Te_3/Bi_2Se_3 film; XPS spectra of (c) Se 3d core level and (d) S 2p core level of the Bi_2Se_3/Bi_2S_3 film; XPS spectra of (e) Te 3d core level and (f) S 2p core level of the Bi_2Te_3/Bi_2S_3 film.

4. UV-Vis absorption spectra

UV-Vis absorption spectroscopy (R1, Ideaoptics) is employed to confirm the light absorption performance of Bi_2Te_3, Bi_2Se_3, and Bi_2S_3 films. The result suggests the Bi_2X_3 shows a high and broad absorption in the visible region. According to the absorption edge in **Figure S4(a-c)**, we can obtain the bandgaps (E_g) of Bi_2X_3 as shown in **Figure 3(b)** in the main text.

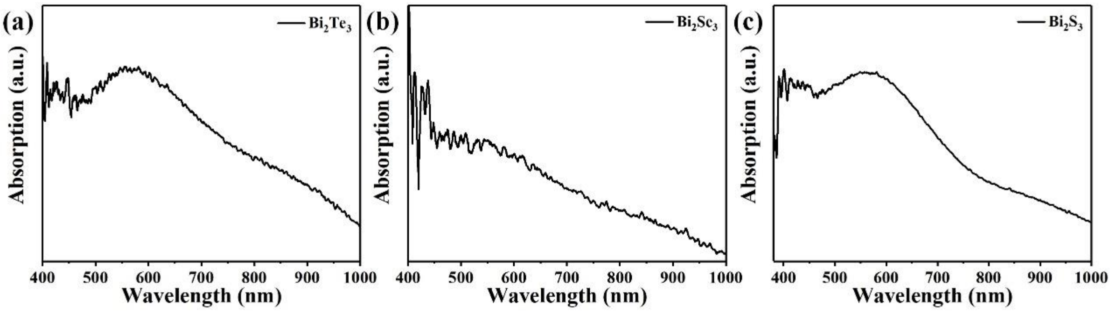

Figure S4. UV-Vis spectra of (a) Bi_2Te_3, (b) Bi_2Se_3, and (c) Bi_2S_3.

5. XPS result of $Bi_2Te_3/Bi_2Se_3/Bi_2S_3$ cascade heterostructure

To confirm the chemical compositions of the $Bi_2Te_3/Bi_2Se_3/Bi_2S_3$ cascade heterostructure, XPS was also measured in **Figure S5**. The Bi 4f core-level spectrum exhibits two main peaks (163.5 and 158.1 eV) from Bi^{3+} as shown in **Figure S5(a)**. Another small peak at 160.9 eV is from Bi metal, which is caused by the thermal decomposition of Bi_2S_3 during the CVD process.

From the S 2p core-level spectrum in **Figure S5(b)**, the peaks of S $2p_{1/2}$ and $2p_{3/2}$ energy levels are observed at the binding energies near 163.5 and 160.1 eV. The four peaks of Te 3d were $3d_{5/2}$ (~572.4 and 576.1 eV) and $3d_{3/2}$ (~582.8 and 586.5 eV) as shown in **Figure S5(c)**. From the XPS spectrum of Se 3d core level in **Figure S5(d)**, Se $3d_{5/2}$ (~54.3 eV) and Se $3d_{7/2}$ (~53.5 eV) belong to Se^{2-} valence state. It is worth noting that the characteristic peaks have an obvious shift toward higher or lower binding energy in these heterostructures those of pure Bi_2Te_3, Bi_2Se_3, and Bi_2S_3 as shown in Figure 1 in the main text. This is due to the interlayer coupling and charge redistribution at the heterostructure interface, which is frequently found in the van der Waals heterostructures. The above-mentioned analysis further confirms the formation of the $Bi_2Te_3/Bi_2Se_3/Bi_2S_3$ film.

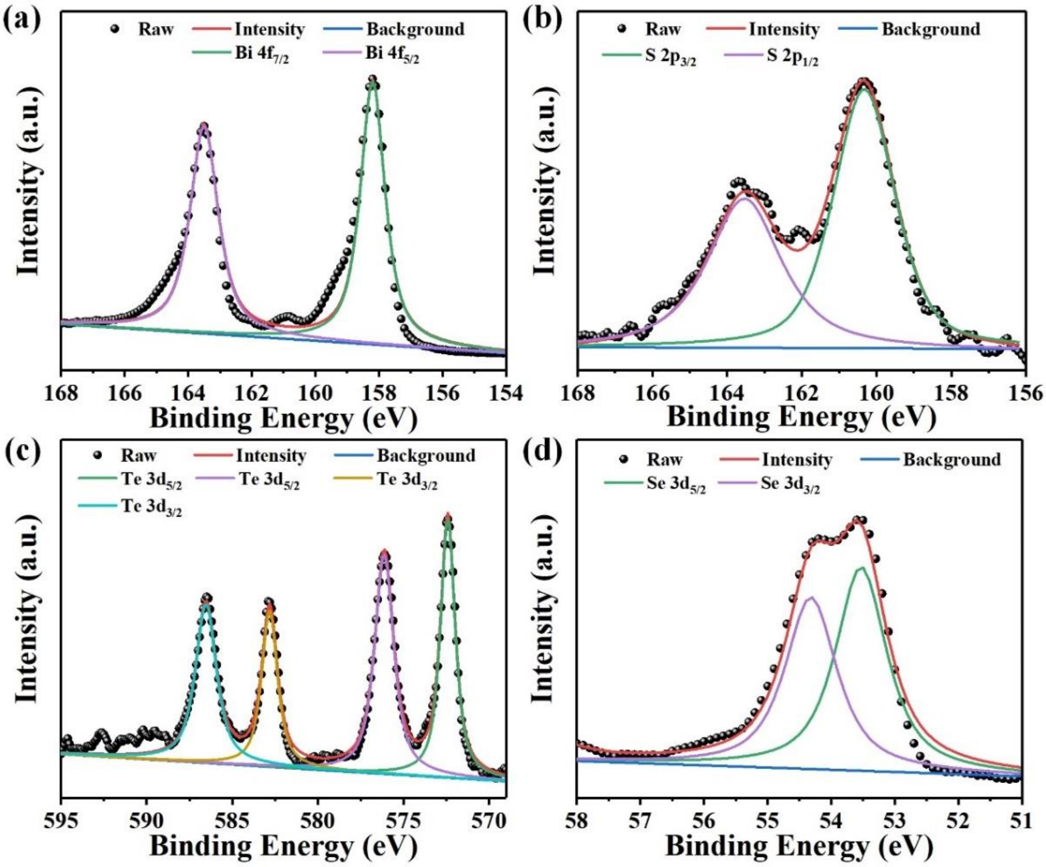

Figure S5. XPS spectra of (a) Bi 4f, (b) S 2p, (c) Te 3d, and (d) Se 3d core levels of the $Bi_2Te_3/Bi_2Se_3/Bi_2S$ film, which are fitted by Gaussian-Lorentz functions.

6. Long-term stability measurement of the $Bi_2Te_3/Bi_2Se_3/Bi_2S_3$ heterostructure

To characterize the stability of PEC-type photodetector, the long-term I-t cyclic stability tests of $Bi_2Te_3/Bi_2Se_3/Bi_2S_3$ heterostructure suggest superior stability within 7200 s at -0.1 V in

Figure S6(a). From the intercepted photocurrent signal in **Figure S6(b-c)**, the photocurrent density keeps almost constant.

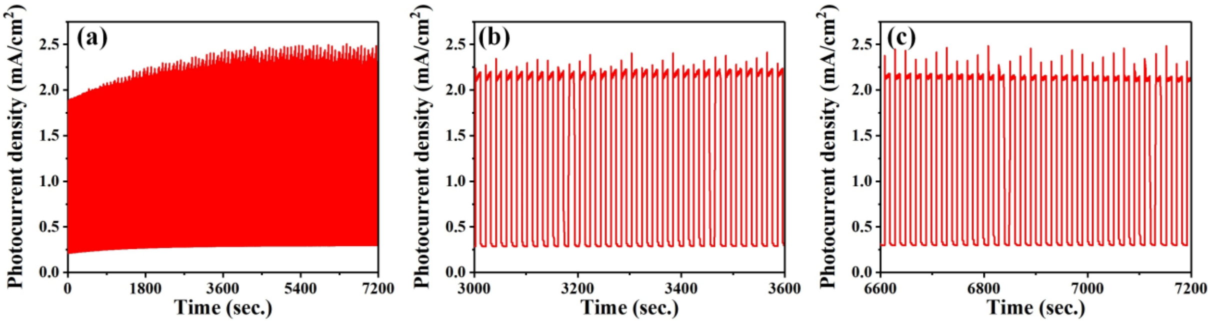

Figure S6. (a) Long-term stability measurement of the $Bi_2Te_3/Bi_2Se_3/Bi_2S_3$ heterostructure at -0.1 V, and the enlarged region of (b) 3000-3600 s and (c) 6600-7200 s

7. Power intensity-dependent photocurrent without a bias voltage

The photocurrent density increases along with the increscent of the incident light irradiation without a bias voltage as shown in **Figure S7**. For instance, the calculated I_{ph} increases from 104 μA cm^{-2} at 50 mW cm^{-2} to 178 μA cm^{-2} at 100 mW cm^{-2} at 0 V.

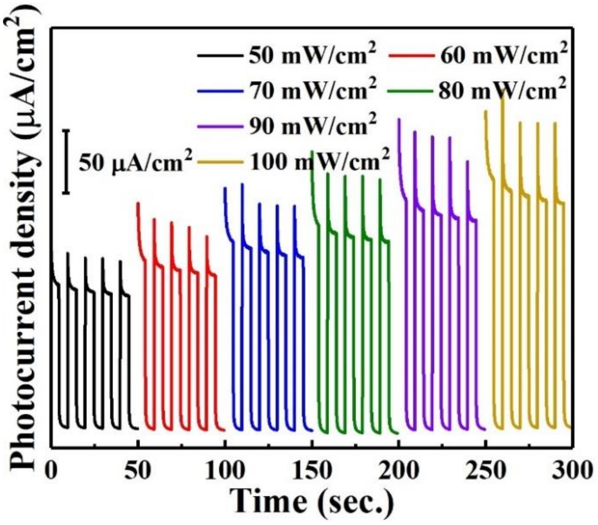

Figure S7. Power intensity-dependent photocurrent without a bias voltage

8. Ultraviolet photoelectron spectroscopy measurements

The charge transfer in the staggered heterostructure can follow different directions such as type-II, Z-scheme and S-scheme mechanism.[4] Principally, the work function ($\Phi = h\nu - E_{cut-off} - E_F$) of semiconductors is an effective way to determine the charge transfer mechanism in heterojunction.[4a] To obtain the Φ values, ultraviolet photoelectron spectroscopy (UPS, Thermo Scientific, ESCALAB Xi$^+$., USA) spectra of Bi_2S_3, Bi_2Se_3, and

Bi_2Te_3 semiconductors were measured as shown in Figure **S8(a)**. In experiment, the photon energy (hν) of the monochromatic He-I source is 21.22 eV. $E_{cut-off}$ and E_F are the secondary electron cut-off edge and Fermi level, respectively. The $E_{cut-off}$ values of Bi_2S_3, Bi_2Se_3, and Bi_2Te_3 are 15.75, 15.58, and 15.46 eV and the corresponding Φ values are 5.47, 5.64, and 5.76 eV, respectively. Based on their staggered heterostructure and Φ values, we plotted the band diagrams of the samples, as shown in Figure **S8(b)**. When Bi_2S_3 and Bi_2Se_3 semiconductors come into contact, the internal free electrons will flow from Bi_2S_3 with a smaller Φ to Bi_2Se_3 with a larger Φ until reaching the Fermi level equilibrium.[4a] Due to the electrostatic induction, Bi_2Se_3 is negatively charged and Bi_2S_3 becomes positively charged at the interface. Thus, a built-in electric field is formed at the interface and the direction of the built-in electric field is from Bi_2S_3 to Bi_2Se_3. Similarly, an internal electric field is also built at the Bi_2Se_3/Bi_2Te_3 interface and the corresponding direction is from Bi_2Se_3 to Bi_2Te_3 as shown in Figure **S8(c)**. The built-in electric field at the Bi_2Te_3/Bi_2Se_3/Bi_2S_3 heterostructure interface facilitates the type-II charge transfer mechanism.

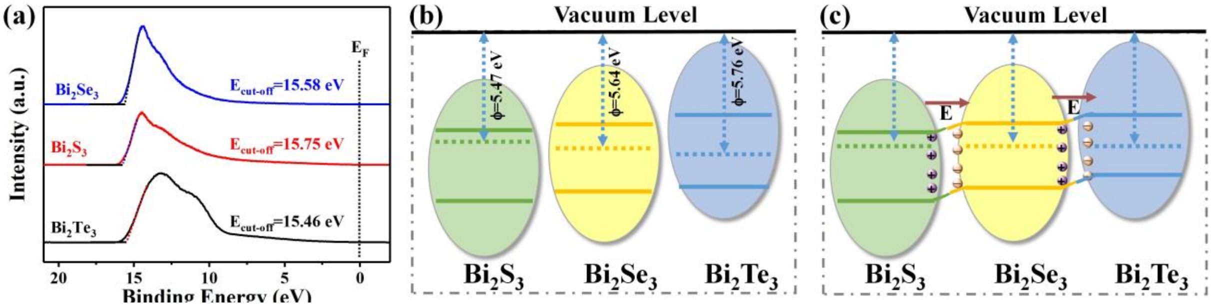

Figure S8 (a) UPS spectra of Bi_2S_3, Bi_2Se_3, and Bi_2Te_3; Energy band structure of Bi_2S_3, Bi_2Se_3, and Bi_2Te_3 (b) before contact and (c) after contact

In the Bi_2Te_3/Bi_2Se_3/Bi_2S_3/ITO heterostructure, the type-II charge transfer pathway facilitates photogenerated electrons transfer to ITO substrate and then toward the Pt cathode for the photocurrent improvement as shown in Figure **S9(a)**. If the charge transfer process follows Z-scheme mode, the photogenerated electrons in Bi_2S_3 would directly recombine with photogenerated holes in Bi_2Se_3. Similarly, the photogenerated electrons in Bi_2Se_3 recombine with photogenerated holes in Bi_2Te_3 as shown in Figure **S9(b)**. This Z-scheme charge transfer process in Bi_2Te_3/Bi_2Se_3/Bi_2S_3/ITO heterostructure greatly impedes the charge transfer toward ITO substrate and then to Pt electrode, thus resulting in the decrease of photocurrent. The

increased photocurrent in the heterostructure further confirms that the $Bi_2Te_3/Bi_2Se_3/Bi_2S_3$ belongs to type-II heterostructure.

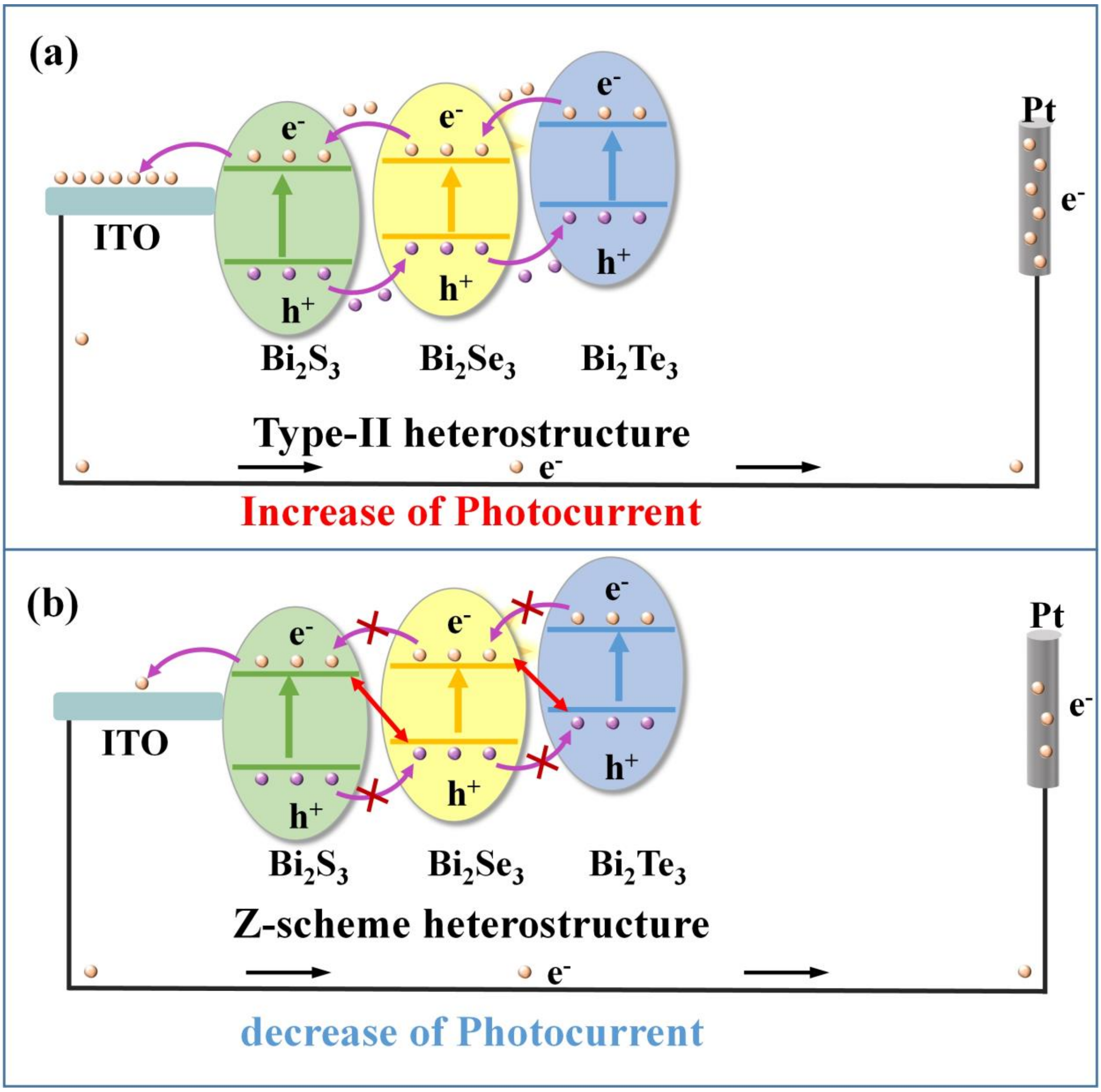

Figure S9 (a) Type-II and (b) Z-scheme charge transfer process in $Bi_2Te_3/Bi_2Se_3/Bi_2S_3$/ITO heterostructure

To exclude the effect of carrier density, the Bi_2S_3/Bi_2Se_3 and Bi_2Se_3/Bi_2S_3 (Bi_2Te_3/Bi_2Se_3 and Bi_2Se_3/Bi_2Te_3) heterostructures were deposited onto ITO substrate under the same growth conditions. The photocurrent was measured as shown in Figure **S10(a-b)** and the photocurrent decreases in the order of Bi_2Se_3/Bi_2S_3 (1047.6 μA cm^{-2}) >Bi_2S_3 (131.5 μA cm^{-2}) >Bi_2S_3/Bi_2Se_3 (95.1 μA cm^{-2}) as shown in Figure **S10(a)** and Bi_2Te_3/Bi_2Se_3 (301.2 μA cm^{-2}) >Bi_2Se_3 (15.9 μA cm^{-2}) >Bi_2Se_3/Bi_2Te_3 (15.0 μA cm^{-2}) as shown in Figure **S10(b)**.

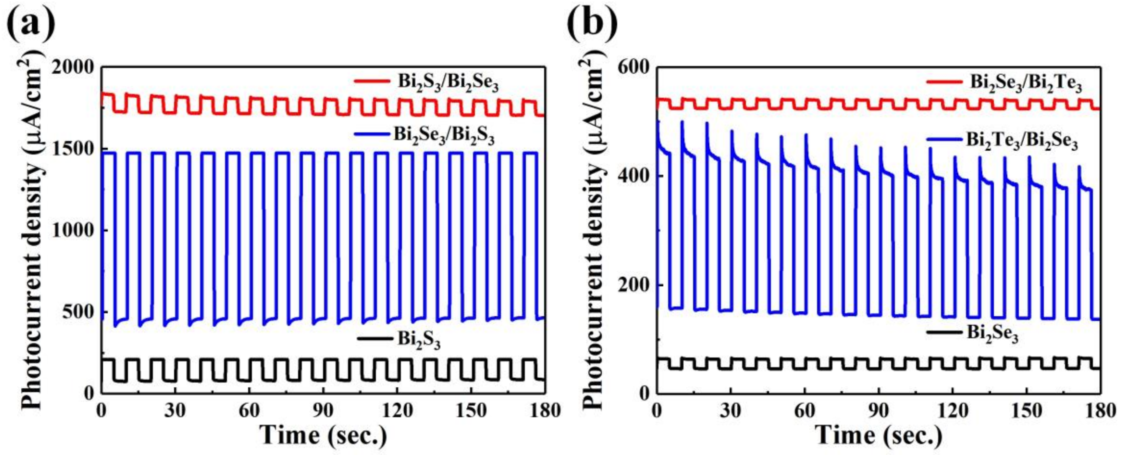

Figure S10 Photocurrent density of (a) Bi_2S_3/Bi_2Se_3, Bi_2Se_3/Bi_2S_3, and Bi_2S_3, and (b) Bi_2Te_3/Bi_2Se_3, Bi_2Se_3/Bi_2Te_3, and Bi_2Se_3

To better understand the charge transfer process, we consider both the type-II and Z-scheme charge transfer processes in the Bi_2Se_3/Bi_2S_3/ITO and Bi_2S_3/Bi_2Se_3/ITO heterostructures are depicted in **Figure S11**. If it is the Z-scheme charge transfer process, the photogenerated electrons in Bi_2S_3 would recombine with the photogenerated holes in Bi_2Se_3 at the heterostructure interface as shown in **Figure S11(a-b)**. This Z-scheme charge transfer process in Bi_2Se_3/Bi_2S_3/ITO heterostructure in **Figure S11(a)** greatly impedes the charge transfer toward ITO substrate and then to Pt electrode, which would result in a lower photocurrent than that of pure Bi_2S_3. Additionally, the photogenerated electrons in Bi_2Se_3 directly transfer toward ITO substrate in **Figure S11(b)**, thus resulting in a higher photocurrent in Z-scheme Bi_2S_3/Bi_2Se_3 heterostructure than that of Bi_2S_3/Bi_2Se_3 heterostructure. However, these results are totally contradictory to our experimental results in **Figure S10(a)**. The results provide solid evidence to confirm that the charge transfer process in Bi_2Se_3/Bi_2S_3 heterostructure cannot follow the Z-scheme type. If the type-II heterostructure is constructed between Bi_2Se_3 and Bi_2S_3, the photogenerated electrons of Bi_2Se_3 quickly transfer toward Bi_2S_3 as shown in **Figure S11(c-d)**. Compared with Bi_2S_3/Bi_2Se_3/ITO heterostructure, much more photogenerated electrons of Bi_2Se_3/Bi_2S_3/ITO heterostructure are collected at ITO substrate and then generate higher photocurrent. This is in consistent with our experimental results in **Figure S10(a)**. Furthermore, the photocurrent of Bi_2S_3 is larger than that of Bi_2S_3/Bi_2Se_3/ITO heterostructure. This is because the photogenerated electrons of Bi_2Se_3 quickly transfer toward Bi_2S_3 and then participate in water reduction reaction as shown in **Figure S11(d)**. The results further confirm that the

Bi_2Se_3/Bi_2S_3 belongs to type-II heterostructure instead of Z-scheme heterostructure. Similarly, the photocurrent measurement (**Figure S10(b)**) also demonstrates that the Bi_2Te_3/Bi_2Se_3 belongs to type-II heterostructure.

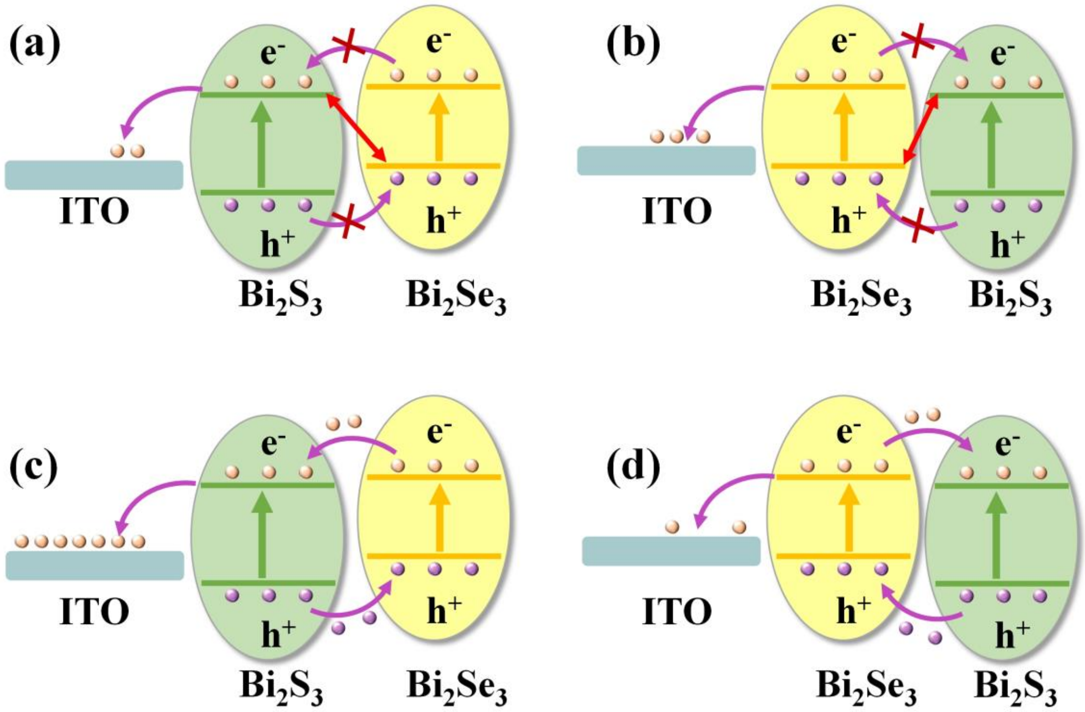

Figure S11 If Z-scheme charge transfer process in (a) Bi_2Se_3/Bi_2S_3/ITO and (b) Bi_2S_3/Bi_2Se_3/ITO heterostructures; If Type-II charge transfer process in (c) Bi_2Se_3/Bi_2S_3/ITO and (d) Bi_2S_3/Bi_2Se_3/ITO heterostructures

Table S1 I_{ph} and R_{ph} values under the wavelengths ≥400 and ≥700 nm

materials	I_{ph} ≥400 (μA cm^{-2})	R_{ph}≥400 nm (mA W^{-1})	I_{ph}≥700 (μA cm^{-2})	R_{ph}≥700 (mA W^{-1})
Bi_2Te_3	13	0.21	0.05	0.0025
Bi_2Se_3	16	0.26	0.2	0.01
Bi_2S_3	119	1.98	5.3	0.265
Bi_2Te_3/Bi_2Se_3	44	0.73	6	0.3
Bi_2Se_3/Bi_2S_3	840	16.0	199	9.95
Bi_2Te_3/Bi_2Se_3	1430	23.8	219	10.95
$Bi_2Te_3/Bi_2Se_3/ Bi_2S_3$	1770	29.5	373	18.65

Table S2 I_{ph} and R_{ph} values under the bias voltage of -0.1 and 0 V

Wavelength (nm)	I_{ph} -0.1 V (μA cm^{-2})	R_{ph} -0.1 V (mA W^{-1})	I_{ph} 0 V (μA cm^{-2})	R_{ph} 0 V (mA W^{-1})
420	902	92.04	34	3.47
450	948	92.03	36	3.50
475	1243	103.58	42	3.5
500	885	88.5	30	3.0
550	1017	78.23	35	2.69
600	604	58.64	24	2.33
650	255	25.76	23	2.32

Table S3. Performance comparison of self-powered PEC photodetectors based on $Bi_2Te_3/Bi_2Se_3/Bi_2S_3$ and other previously reported advanced materials

Material	Type	Light	I_{ph} level	R_{ph} (mA W^{-1})	t_{res}/t_{rec} (ms)	Ref
$Bi_2Te_3/Bi_2Se_3/Bi_2S_3$	PEC (0.1 mol L^{-1} Na_2S/0.02 mol L^{-1} Na_2SO_3)	475 nm	μA/cm^2	3.5	8/8	This work
AlGaN	(0.5 M H_2SO4)	254 nm	μA/cm^2	3	163/15	[5]
Bi	PEC (1 M KOH)	350 nm	nA/cm^2	0.0193	100/200	[6]
Bi_2S_3	PEC (0.1 M KOH)	365 nm	nA/cm^2	0.0089	100/100	[7]
Bi_2O_2S	PEC (1.0 M KOH)	365 nm	μA/cm^2	2.4	30/50	[8]
Ga_2O_3	PEC (0.5 M Na_2SO_4)	254 nm	μA/cm^2	3.81	290/160	[9]
ZnS	PEC (I_3^-/I^-)	265 nm	μA/cm^2	19.4	250/200	[10]
BP	PEC (0.1 M Na_2SO_4)	Sun	nA/cm^2	0.002	500/500	[11]
Te@Bi	PEC (0.5 M KOH)	Sun	μA/cm^2	0.03	80/80	[12]
TiO_2/ZnO	PEC (I_3^-/I^-)	265 nm	μA/cm^2	16	13.5/4	[13]
InSe/Ge-InSe	PEC (0.1 M KOH)	Sun	nA/cm^2	0.003	95/91	[14]

Table S4 The D^*, EQE, and IPCE values under the bias voltage of -0.1 and 0 V

Wavelength (nm)	D^* -0.1 V (10^9 Jones)	D^* -0 V (10^9 Jones)	EQE -0.1 V (%)	EQE 0 V (%)	IPCE -0.1 V (%)	IPCE 0 V (%)

420	8.26	1.74	26.30	0.99	24.03	0.91
450	8.01	1.91	24.54	0.93	24.03	0.91
475	8.96	1.99	26.17	0.88	27.04	0.91
500	7.32	1.75	21.24	0.72	23.10	0.78
550	6.52	1.58	17.07	0.59	20.42	0.70
600	4.74	1.40	11.73	0.47	15.31	0.61
650	1.90	1.59	4.76	0.43	6.72	0.61

Table S5 I_{ph} and R_{ph} values under different power intensity of -0.1 and 0 V

Power intensity (mW cm^{-2})	I_{ph} -0.1 V (μA cm^{-2})	R_{ph} -0.1 V (mA W^{-1})	I_{ph} 0 V (μA cm^{-2})	R_{ph} 0 V (mA W^{-1})
50	1130	22.6	104	2.08
60	1380	23.0	121	2.02
70	1590	22.7	135	1.93
80	1850	23.1	148	1.85
90	2060	22.9	165	1.83
100	2390	23.9	178	1.78

Table S6. PEC hydrogen production of Bi_2X_3 and related heterostructures

Materials	Light	Electrolyte	Bias voltage	H_2 rate (μmol/cm^2/h)	Ref
Bi_2Te_3	visible	0.1 mol L^{-1} Na_2S and 0.02 mol L^{-1} Na_2SO_3	-0.1 V vs Ag/AgCl	36	This work
Bi_2Se_3				92	
Bi_2S_3				136	
Bi_2Te_3/Bi_2Se_3				172	
Bi_2Se_3/Bi_2S_3				204	
Bi_2Te_3/Bi_2Se_3				332	
$Bi_2Te_3/Bi_2Se_3/Bi_2S_3$				416	
$Bi_2Te_3/Bi_2Se_3/Bi_2S_3$		water		14.32	

Bi_2Se_3		0.1 M Na_2SO_4		184	
TiO_2/Bi_2Se_3				261	
Bi_2Se_3		0.1 M Na_2SO_3 and Na_2S	1.23 V vs RHE	658	
TiO_2/Bi_2Se_3	1 sun			1081	[15]
$Bi_2Te_3/V_{0.04}$-Sb_2Te_3	-	0.5 M H_2SO_4	J=10 mA cm^{2}	170	[16]
V-Bi_2S_3 nanowires	visible	0.1 M Na_2SO_3 and 0.1 M Na_2S	-0.1 V vs Ag/AgCl	70	[17]
Bi_2S_3 nanowires				60	
$Bi_2S_3/BiVO_4$	1 sun	0.2 M Na_2SO_3	0.45 V vs RHE	33.4	[18]
Bi_2S_3-$BiOBr/TiO_2$	visible	0.25 mol L^{-1} Na_2S and 0.35 mol L^{-1} Na_2SO_3	-	23.75	[19]
$Bi_2S_3/BiVO_4$	1 sun	0.1 M Na_2S and 0.02 M Na_2SO_3	0.67 V vs. RHE	417	[20]
Bi_2S_3@TiO_2	visible	0.25 mol L^{-1} Na_2S and 0.35 mol L^{-1} Na_2SO_3	-0.2 V vs Ag/AgCl	35.97	[21]

References:

[1] Y.-F. Lin, H.-W. Chang, S.-Y. Lu, C. W. Liu, *J. Phys. Chem. C* **2007**, *111*, 18538.
[2] A. Subrati, Y. Kim, Y. Al Wahedi, V. Tzitzios, S. Alhassan, H. J. Kim, S. Lee, E. Sakellis, N. Boukos, S. Stephen, S. M. Lee, J. B. Lee, M. Fardis, G. Papavassiliou, *CrystEngComm* **2020**, *22*, 7918.
[3] C. Lu, M. Luo, D. Yang, J. Ma, M. Qi, X. Xu, *Adv. Mater. Interfaces* **2021**, *8*, 2100913.
[4] a)S. Sun, R. Gao, X. Liu, L. Pan, C. Shi, Z. Jiang, X. Zhang, J.-J. Zou, *Sci. Bull.* **2022**, *67*, 389; b)K. Alkanad, A. Hezam, Q. A. Drmosh, S. S. Ganganakatte Chandrashekar, A. A. AlObaid, I. Warad, M. A. Bajiri, L. Neratur Krishnappagowda, *Sol. RRL* **2021**, *5*, 2100501; c)S. M. Ghoreishian, K. S. Ranjith, B. Park, S.-K. Hwang, R. Hosseini, R. Behjatmanesh-Ardakani, S. M. Pourmortazavi, H. U. Lee, B. Son, S. Mirsadeghi, Y.-K. Han, Y. S. Huh, *Chem. Eng. J.* **2021**, *419*, 129530; d)C. Cheng, B. He, J. Fan, B. Cheng, S. Cao, J. Yu, *Adv. Mater.* **2021**, *33*, 2100317.
[5] D. Wang, X. Liu, S. Fang, C. Huang, Y. Kang, H. Yu, Z. Liu, H. Zhang, R. Long, Y. Xiong, Y. Lin, Y. Yue, B. Ge, T. K. Ng, B. S. Ooi, Z. Mi, J.-H. He, H. Sun, *Nano Lett.* **2021**, *21*, 120.
[6] C. Xing, W. Huang, Z. Xie, J. Zhao, D. Ma, T. Fan, W. Liang, Y. Ge, B. Dong, J. Li, H. Zhang, *ACS Photonics* **2018**, *5*, 621.
[7] W. Huang, C. Xing, Y. Wang, Z. Li, L. Wu, D. Ma, X. Dai, Y. Xiang, J. Li, D. Fan, H. Zhang, *Nanoscale* **2018**, *10*, 2404.

[8] X. Yang, L. Qu, F. Gao, Y. Hu, H. Yu, Y. Wang, M. Cui, Y. Zhang, Z. Fu, Y. Huang, W. Feng, B. Li, P. Hu, *ACS Appl. Mater. Interfaces* **2022**, *14*, 7175.
[9] K. Chen, S. Wang, C. He, H. Zhu, H. Zhao, D. Guo, Z. Chen, J. Shen, P. Li, A. Liu, C. Li, F. Wu, W. Tang, *ACS Appl. Nano Mater.* **2019**, *2*, 6169.
[10] D. Li, S. Hao, G. Xing, Y. Li, X. Li, L. Fan, S. Yang, *J. Am. Chem. Soc.* **2019**, *141*, 3480.
[11] X. Ren, Z. Li, Z. Huang, D. Sang, H. Qiao, X. Qi, J. Li, J. Zhong, H. Zhang, *Adv. Funct. Mater.* **2017**, *27*, 1606834.
[12] Y. Zhang, F. Zhang, L. Wu, Y. Zhang, W. Huang, Y. Tang, L. Hu, P. Huang, X. Zhang, H. Zhang, *Small* **2019**, *15*, 1903233.
[13] Y. Wang, L. Chen, H. Zhou, K. Wei, Z. Zhu, E. Xie, W. Cao, W. Han, *J. Mater. Chem. C* **2019**, *7*, 8011.
[14] L. Liao, B. Wu, E. Kovalska, F. M. Oliveira, J. Azadmanjiri, V. Mazánek, L. Valdman, L. Spejchalová, C. Xu, P. Levinský, J. Hejtmánek, Z. Sofer, *Nanoscale* **2022**, *14*, 5412.
[15] P. Subramanyam, B. Meena, D. Suryakala, C. Subrahmanyam, *ACS Appl. Nano Mater.* **2021**, *4*, 739.
[16] Q. Wang, L. Huang, Y. Wu, G. Ma, Z. Lei, S. Ren, *Int. J. Hydrogen Energy* **2022**, *47*, 21361.
[17] Y.-Y. Geng, C.-L. Tao, S.-F. Duan, J. San Martin, Y. Lin, X. Zhu, Q.-Q. Zhang, X.-W. Kang, S.-S. He, Y.-X. Zhao, X. Li, L. Niu, D.-D. Qin, Y. Yan, D.-X. Han, *Catal. Today* **2020**, *350*, 47.
[18] F. Li, D. Y. C. Leung, *Chem. Eng. Sci.* **2020**, *211*, 115266.
[19] Y. Jia, P. Liu, Q. Wang, Y. Wu, D. Cao, Q.-A. Qiao, *J. Colloid Interface Sci.* **2021**, *585*, 459.
[20] M. A. Mahadik, H.-S. Chung, S.-Y. Lee, M. Cho, J. S. Jang, *ACS Sustainable Chem. Eng.* **2018**, *6*, 12489.
[21] C. Liu, Y. Yang, W. Li, J. Li, Y. Li, Q. Chen, *Chem. Eng. J.* **2016**, *302*, 717.
